# Immediate and Delayed Effects of Joint Loading Activities on Knee and Hip Cartilage: A Systematic Review and Meta-analysis

**DOI:** 10.1186/s40798-023-00602-7

**Published:** 2023-07-14

**Authors:** Sally L. Coburn, Kay M. Crossley, Joanne L. Kemp, Stuart J. Warden, Tom J. West, Andrea M. Bruder, Benjamin F. Mentiplay, Adam G. Culvenor

**Affiliations:** 1 La Trobe Sport and Exercise Medicine Research Centre, School of Allied Health, Human Services and Sport, La Trobe University, Melbourne, VIC Australia; 2 Department of Physical Therapy, School of Health & Human Sciences, Indiana University, Indianapolis, IN USA

**Keywords:** Articular cartilage, Magnetic resonance imaging, Activity, Joint loading

## Abstract

**Background:**

The impact of activity-related joint loading on cartilage is not clear. Abnormal loading is considered to be a mechanical driver of osteoarthritis (OA), yet moderate amounts of physical activity and rehabilitation exercise can have positive effects on articular cartilage. Our aim was to investigate the immediate effects of joint loading activities on knee and hip cartilage in healthy adults, as assessed using magnetic resonance imaging. We also investigated delayed effects of activities on healthy cartilage and the effects of activities on cartilage in adults with, or at risk of, OA. We explored the association of sex, age and loading duration with cartilage changes.

**Methods:**

A systematic review of six databases identified studies assessing change in adult hip and knee cartilage using MRI within 48 h before and after application of a joint loading intervention/activity. Studies included adults with healthy cartilage or those with, or at risk of, OA. Joint loading activities included walking, hopping, cycling, weightbearing knee bends and simulated standing within the scanner. Risk of bias was assessed using the Newcastle–Ottawa Scale. Random-effects meta-analysis estimated the percentage change in compartment-specific cartilage thickness or volume and composition (T2 relaxation time) outcomes. The Grading of Recommendations Assessment, Development and Evaluation (GRADE) system evaluated certainty of evidence.

**Results:**

Forty studies of 653 participants were included after screening 5159 retrieved studies. Knee cartilage thickness or volume decreased immediately following all loading activities investigating healthy adults; however, GRADE assessment indicated very low certainty evidence. Patellar cartilage thickness and volume reduced 5.0% (95% CI 3.5, 6.4, *I*^2^ = 89.3%) after body weight knee bends, and tibial cartilage composition (T2 relaxation time) decreased 5.1% (95% CI 3.7, 6.5, *I*^2^ = 0.0%) after simulated standing within the scanner. Hip cartilage data were insufficient for pooling. Secondary outcomes synthesised narratively suggest knee cartilage recovers within 30 min of walking and 90 min of 100 knee bends. We found contrasting effects of simulated standing and walking in adults with, or at risk of, OA. An increase of 10 knee bend repetitions was associated with 2% greater reduction in patellar thickness or volume.

**Conclusion:**

There is very low certainty evidence that minimal knee cartilage thickness and volume and composition (T2 relaxation time) reductions (0–5%) occur after weightbearing knee bends, simulated standing, walking, hopping/jumping and cycling, and the impact of knee bends may be dose dependent. Our findings provide a framework of cartilage responses to loading in healthy adults which may have utility for clinicians when designing and prescribing rehabilitation programs and providing exercise advice.

**Supplementary Information:**

The online version contains supplementary material available at 10.1186/s40798-023-00602-7.

## Key Points

- Weightbearing activities seem to cause minimal change in knee cartilage of healthy adults.
- Immediate knee cartilage changes after weightbearing activities tend to recover within 15–90 min in healthy adults.
- Patellar cartilage seems to be particularly affected by loaded knee bends, with the magnitude of cartilage change related to the number of repetitions performed.

## Background

Abnormal joint loading is considered a key mechanical driver of osteochondral changes thought to contribute to the initiation and progression of knee and hip osteoarthritis (OA) [1]. It is not clear what intensity or type of loading may increase OA risk as under-/overloading can result in diminution of cartilage thickness and volume and compositional biomarkers [2–5], but moderate physical activity programs [4, 6] and rehabilitation exercises for OA [7], and knee surgery [8] can have positive effects on cartilage composition. Determining the effect(s) of a known dose of load on knee and/or hip cartilage is difficult to establish over an extended period due to the influence of potential confounding factors (e.g. occupational workloads, injury, body mass index [BMI], levels of compliance/drop out with exercise interventions) [9]. Alternatively, exploring the immediate and delayed effects (within 48 h) of loading on knee and hip cartilage allows for tight control of activity parameters and evaluation of the impact of potential confounders.

We recently synthesised data evaluating the immediate effects of running on cartilage assessed using magnetic resonance imaging (MRI) and found small (likely transient) reductions in knee cartilage thickness and volume (declines of 3–5%) and composition (declines of 4–13%) in healthy adults [10], similar to prior systematic reviews [11–14]. The immediate and delayed effects of joint loading activities, such as those commonly recommended to meet physical activity guidelines or achieve rehabilitation goals (e.g. walking, cycling, squatting), have not been synthesised using meta-analysis. Quantifying MRI cartilage changes in response to activity in people with healthy knees and those with, or at risk of, OA could inform our understanding of optimal loading for individuals to meet physical activity guidelines and following injury, to reduce OA risk and to design therapeutic exercise programs that facilitate cartilage health.

The primary aim of this study was to investigate the immediate effect of joint loading activities other than running on hip and/or knee articular cartilage, as evaluated with MRI, in healthy adults. The secondary aims were to investigate: the delayed (20 min–48 h) effects of joint loading activities, the effects of activities on cartilage in adults with, or at risk of, OA and the explore associations between cartilage changes and sex, age, and loading duration/repetitions.

## Methods

### Protocol and Registration

The systematic review protocol was prospectively registered (PROSPERO, CRD 42020209368) as part of a larger protocol investigating the immediate effect of various joint loading activities on knee and/or hip cartilage [10]. The study adheres to the Preferred Reporting Items for Systematic Reviews and Meta-Analyses (PRISMA) guidelines [15].

### Search Strategy

A systematic search of Medline, Embase, and Cochrane Central Register of Controlled Trials (via Ovid), CINAHL and SPORTDiscus (via EBSCOhost), and Web of Science (via Clarivate) databases, with no restriction of publication year or language, was conducted in July 2020. The original and updated searches (in June 2021 and April 2023) used a search strategy customised for each database that included Medical Subject Heading (MeSH) terms and text words in title, abstract and as keywords related to four key themes: knee/hip and associated injuries, exercise, MRI and cartilage (Additional file 1).

Two authors (SC and TW) identified eligible studies by independently screening the title, abstract and relevant full text. Eligible study reference lists were searched recursively until no additional eligible publications were identified. Disagreement regarding eligibility was resolved by discussion. A third reviewer (AC) was available if consensus could not be reached.

### Eligibility Criteria

Peer-reviewed studies were eligible for inclusion if they used any MRI measures of cartilage thickness, volume or composition to investigate changes in knee or hip articular cartilage in individuals with mean age ≥ 18 years (as immature cartilage may respond differently to mechanical load [16, 17]), with at least one scan performed within 48 h prior to and following a joint loading intervention/activity. Due to the large number of studies retrieved, the effects of running were synthesised separately and reported elsewhere [10].

To achieve our primary aim, studies for the current review investigated the effect of any joint loading activity other than running in adults with healthy joints. We also included studies that investigated individuals with, or at risk of developing OA (i.e. high BMI, post-anterior cruciate ligament [ACL] injury or surgery, with femoroacetabular impingement) to achieve our secondary aims. Joint loading activities were defined as any hip or knee joint loading exercise or physical activity that was intentional, land-based and comprised of planned, structured movement or activity (e.g. walking, hopping, weightbearing knee bends and included simulated standing within the scanner) of any type, duration or intensity.

Cartilage changes measured in the same individuals following a second joint loading activity were included if pre-/post-activity MRI was performed at each instance and the activities were separated by > 1 week (to limit potential confounding of cartilage changes that may be detected within 48 h following a strenuous activity [18]), reflecting methodology used in likely eligible studies identified during review development [19]. We excluded non-English language, non-original data studies, case reports, studies of animals or cadavers and studies of other rheumatological diseases. We excluded studies without available full-text or with incomplete data if authors were unable to provide data when contacted.

### Risk of Bias

Risk of bias was assessed independently by two authors (SC, AB), using a modified version of the Newcastle–Ottawa Scale (NOS) [20, 21] (Additional file 2). Relevant items were adapted from the NOS cohort and case–control study scales to assess selection bias (i.e. inclusion criteria, representativeness, sample size), participant comparability (i.e. control of activity prior to baseline MRI, cohort comparability) and observation bias (i.e. MRI assessor blinding, MRI assessor qualifications and reliability, MRI outcome reliability/validity and follow-up adequacy). Risk of bias was rated low, high or not applicable for each of the nine items and considered to be low risk of bias overall if more than half of applicable items (i.e. ≥ 5/8 or ≥ 5/9) were rated low risk. Completed appraisals were discussed in a consensus meeting, and disagreements were decided by an independent arbitrator (JK). Cohen’s Kappa (K) was calculated to assess agreement between raters.

### Data Extraction

Data were extracted by one reviewer (SC), recorded in a customised spreadsheet and cross-checked by two reviewers (AC and BM). Participant characteristics (e.g. sex, age, BMI, joint status [e.g. healthy or at risk of/having OA]) together with number of participants/joints and joint loading activity characteristics were extracted. MRI data extracted included MRI sequences utilised and percent change in MRI thickness or volume and/or compositional outcomes. Semiquantitative outcomes included scores of defect size and severity such as the Scoring Hip Osteoarthritis with MRI (SHOMRI) cartilage sub-score for the hip [22] or the Whole-Organ MRI Score (WORMS) cartilage sub-score for the knee [23]. Compositional outcomes included specialised MRI techniques used to provide measures of cartilage composition (considered to be biomarkers of early OA [24, 25]), e.g. T1ρ, T2 and T2* relaxation times, and T1-delayed gadolinium-enhanced MRI of cartilage (dGEMRIC) index. Relaxation time measures are increased when loss of matrix integrity results in decreased concentration of collagen or proteoglycan components and increased or altered distribution of cartilage hydration [26, 27]. Hence, higher relaxation times reflect poorer cartilage health. Longer T1 dGEMRIC relaxation time, measured after injection of a contrast agent, is indicative of higher glycosaminoglycan content and therefore better cartilage quality [28].

Data that documented change in cartilage outcomes from scans conducted closest to the joint loading activity were extracted to achieve our primary aim and explore the immediate effects of joint loading activities. Data from studies that repeated measures > 20 min but within 48 h of activity completion, in the same individuals, were extracted to achieve one of our secondary aims to investigate the delayed effects of activities. For studies that measured the effects of an activity at different intensities (e.g. walking 10 min and walking 60 min on separate occasions), we selected data from the activity that was most similar in intensity to other studies in the analysis. Authors were contacted for data if results were presented graphically or bilaterally. If authors were unable to provide the requested data, a website tool (WebPlotDigitizer, version 4.5, Pacifica, USA) was used to obtain data from graphed results and bilateral data were reported narratively.

### Data Synthesis and Analysis

The primary outcome was the percent change in mean MRI cartilage measures from scans performed before and immediately (i.e. within 20 min) after a joint loading activity, because significant effects of loading can occur a short time after activity completion [12]. Delayed measures of cartilage changes (21 min to 48 h after activity) were pooled if data from scans with comparable timing were available, with 48 h chosen as it is the time period where cartilage may be sensitive to a loading event [18, 29]. The equation $\frac{{\left( {\text{post-load mean}} \right) - \left( {\text{pre-load mean}} \right)}}{{\text{pre-load mean}}} \times 100$ calculated the percent change from pre- and post-joint loading activity means when data were not presented as percent change from baseline. A Taylor expansion equation [30] and a correlation coefficient (correlation = 0.9, derived from an included study with raw and percentage change data) [31], were used to estimate the standard deviation (SD) of the percentage change, as recommended in the Cochrane Handbook [32].

Study results were pooled using random effects meta-analyses and restricted maximum likelihood (REML) models [33] displayed as forest plots (Stata SE 17 *metan* command), based on the most commonly reported cartilage regions in the knee (i.e. weightbearing femoral, tibial, patellar, femoral trochlear) and hip (i.e. weightbearing femoral, weightbearing acetabular). Data from studies that reported mean percent change in smaller sub-regions (e.g. superficial, deep) were combined according to methods for combining means and SD in the Cochrane Handbook [32]. We synthesised measures of cartilage thickness and volume together with a preference for volume if both measures were reported in the same study as volume has superior reproducibility for the detection of cartilage changes over time [34]. If the same study reported more than one compositional outcome, we selected the single outcome to synthesise using the following hierarchy (according to frequencies found in our previous study [10] and expert opinion [35]): T2, T1ρ, T2* and T1-dGEMRIC. Heterogeneity was calculated for each meta-analysis using the *I*^2^ statistic (where 100% is maximal inconsistency) to quantify the impact of inconsistency between studies [36].

Narrative synthesis was used to report results from studies that could not be pooled, such as bilateral and semi-quantitative measures of cartilage change and studies reporting incomparable cartilage regions, loading activities or timing of MRI measures. Meta-regression analysis explored associations between study level characteristics (i.e., sex, age, activity duration/repetitions) and changes in MRI cartilage thickness and volume.

### Certainty of the Evidence

The Grading of Recommendations Assessment, Development and Evaluation (GRADE) system appraised the overall certainty in the pooled body of evidence using five criteria: risk of bias, consistency of the reported results, indirectness of evidence, imprecision and publication bias [37]. Funnel plot symmetry and Egger tests for small-study effects were used to analyse publication bias in meta-analyses that contained ≥ 10 studies [38].

## Results

### Study Characteristics

**Fig. 1:**
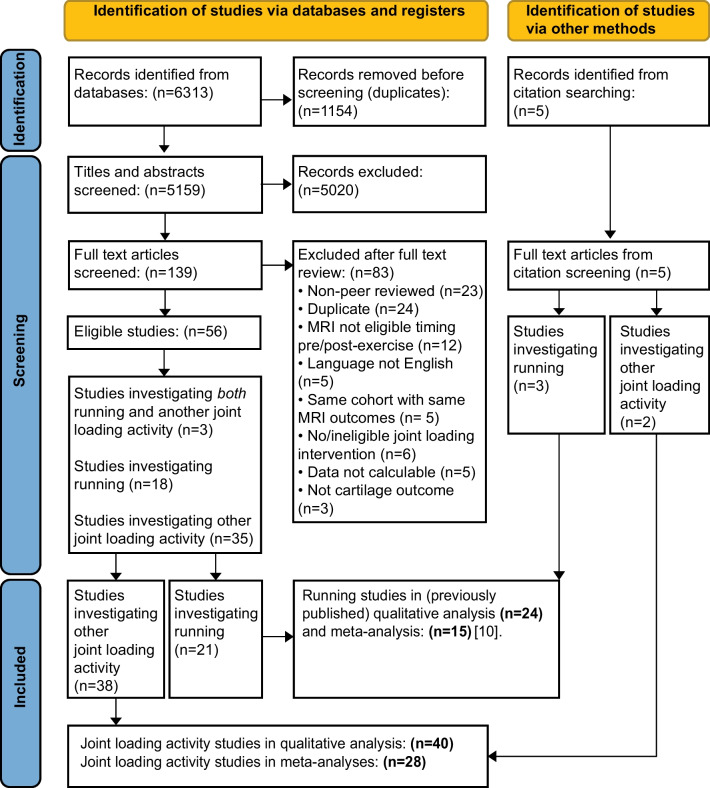
PRISMA flow chart of included studies

Thirty-eight studies of the knee (*n* = 647 participants, 40% female) and two studies of the hip (*n* = 33 participants, 72% female) were included (Fig. 1, Table 1). Participants with healthy knees (*n* = 511) were predominantly young adults (mean age 31 ± 15 years) with a healthy BMI (mean 23 ± 5 kg m^−2^). Participants with, or at risk of, knee OA (*n* = 168) were typically older (mean age 47 ± 12 years) and overweight (mean BMI 27 ± 23 kg m^−2^). Participants with healthy hips (*n* = 18) were young adults (mean age 30 years, range 27–33) with a healthy BMI (mean 22 kg m^−2^, range 21–22), while participants at risk of hip OA (*n* = 9) were older (mean age 39 years, range 24–50) with a healthy BMI (mean 22 kg m^−2^, range 19–30).

**Table 1:** Included studies

Study	Participants (*n*)	Mean age (SD) [range]	Mean BMI (SD) [range]	Female (%)	Selection criteria	Eligible joint loading activity^b^	Eligible joint loading intensity^b^	Eligible joint load duration (min)^b^	Eligible MRI outcome pre- & post-loading activity^b^	MRI Tesla (T)	ROI (*n*)^b^	Eligible comp-artment^b^
Chen et al. [65]^a^	23	25 [23–30]	20.3 [16–24.8]	52	Healthy adults 23–30 yrs	Walk	6.1 km/hr	30	T2 relaxation time, T2* relaxation time	3.0T	23	Patellar Tibial Femoral
Collins et al. [39]	15 Healthy BMI: 8 High BMI: 7	Healthy BMI: 30 [22–45] High BMI: 32 [22–45]	Healthy BMI: 22.8 [18–25] High BMI: 32.8 [30–36]	Healthy BMI: 38 High BMI: 57	No history lower limb injury/surgery/OA symptoms	Walk	Fr = 0.25 (mean 5.47 km/hr)	20	Thickness	3.0T	2	Tibial Femoral
Cotofana et al. [40]	30 Healthy: 11 OA-KL2: 10 OA-KL3: 9	Healthy: 52 (7) OA-KL2: 56 (5) OA-KL3: 58 (4)	NR	100	Healthy: KL0; no history pain/stiff and WOMAC = 0 OA-KL2 or 3: no history surgery or injection	Simulated stand within scanner	50% BW	45	Thickness	3.0T	4	Tibial Femoral
Crook et al. [41]	8	35 [22–48]	24.8 [21.9–27.9]	25	Healthy knee & ACLD knee: No history other knee injury/surgery/OA symptoms/FT cartilage defect/meniscal tear	Walk	4.0 km/hr	20	Thickness	3.0T	4	Tibial Femoral
Cutcliffe et al. [42]	11	25 [22–32]	22.1 [19.8–24.9]	55	No history lower limb injury/smoking	Walk	Fr = 0.25 (mean 5.47 km/hr)	30	Thickness	3.0T	1	Tibial
Eckstein et al. [45]	7	[23–32]	NR	43	Healthy	Knee bends	100 reps (90° knee flexion)	3	Volume	1.5T	1	Patellar
Eckstein et al. [44]	12	25 [22–30]	NR	50	Healthy	Knee bends	Knee bends: 30 reps (120° knee flexion)	1	Volume	1.5T	1	Patellar
Eckstein et al. [43]	50 Gp 1: 12 Gp 2: 10 Gp 3: 14 Gp 4: 7 Gp 5: 7	Gp 1: 25 [22–30] Gp 2: [18–37] Gp 3: 25 (2) Gp 4: 23 (3) Gp 5: 29 (4)	NR	Gp 1: 43 Gp 2: 50 Gp 3: 0 Gp 4: 0 Gp 5: 0	Gp 1: NR Gp 2: NR Gp 3: Untrained Gp 4: Weightlifters Gp 5: Bobsleigh sprinters	Gp 1: Walk, run, cycle Gp 2: Single-leg knee bends, Single leg drop jumps Gp 3–5: Knee bends	Gp 1:NR Walk: level Run: 200 m Cycle: 80 Hz Gp 2: Single leg knee bends: 30 reps, (120° knee flexion), 200% BW Single leg drop jumps: 10 reps (from 40 cm height) Gp 3–5: Single leg knee bends: 30 reps, (120° knee flexion)	Gp 1: Walk: 5 Run: NR Cycle: 10 Gp 2: Single leg knee bends:1 Single leg drop jumps: NR Gp 3–5: 1	Volume	1.5T	Gp 1: 1 Gp 2: 4 Gp 3–5: 1	Gp 1: Patellar Gp 2: Tibial Femoral Gp 3–5: Patellar
Farrokhi et al. [46]	20 Healthy: 10 PFP: 10	Healthy: 27 (4) PFP: 28 (4)	NR	100	Healthy: age/height/weight-matched with no history knee pain/surgery/dislocation/neurological disorder PFP: retropatellar pain of insidious onset & no history peripatellar pain/knee surgery/dislocation/neurological disorder	Knee bends	50 reps (110°) plus 25% BW	1.8	Thickness, T2 relaxation time	3.0T	2	Patellar
Gatti et al. [47]^1^	15	26 (4)	23.7 (2.6)	0	Healthy men	Cycle	80 rpm	46	Thickness, volume, T2 relaxation time	3.0T	4	Tibial Femoral
Hatcher et al. [66]^3^	6	NR	NR	NR	NR	NR	4.0 km/hr	20	T1ρ relaxation time	3.0T	2	Medial & lateral tibiofemoral
Hesper et al. [76]	9	27 (4)	22.9 (1.6)	44	Asymptomatic volunteers	Squats	50 reps	NR	T2* relaxation time	3.0T	2	Hip
Ho et al. [48]	19 Healthy: 10 OA: 9	Healthy: 55 (2) OA: 56 (5)	Healthy: 25.5 (3.0) OA: 32.4 (4.4)	Healthy: 50 OA: 44	Adults 50–65 yrs	Walk	4.8–6.4 km/hr	30	Thickness	3.0T	2	Femoral
Horng et al. [19]	10	23 (1)	22.0 (2.0)	50	Healthy volunteers; no elite athletes, no trauma/chronic disease requiring immobilisation, no previous knee pain/surgery	Knee bends	50 reps (> 90° knee flexion)	10	Thickness	3.0T	4	Patellar Tibial Femoral
Hudelmaier et al. [49]	23	F: 61 (10) M: 60 (5)	NR	52	Asymptomatic subjects 50–78 yrs; no history knee surgery/pain/trauma	Knee bends	30 reps (120° knee flexion)	NR	Thickness	1.5T	1	Patellar
Jogi et al. [50]	9	NR	NR	0	Healthy no history knee injury or pathology	Simulated stand supine in scanner	50% BW	NR	Thickness	3.0T	4	Tibial Femoral
Lad et al. [51]	8	25 (22–30)	22.3 (20.2–25.1)	50	Healthy adults no history injury/surgery either knee	Walk	1.1 m/s	20	Thickness	3.0T	4	Tibial Femoral
Lange et al. [67]	9	27 (2)	NR	0	Healthy subjects	Simulated squat within scanner	20 kg (40° knee flexion)	NR	T2 relaxation time, T1ρ relaxation time	3.0T	1	Patellar
Lange et al. [52]	14	29 (2)	23.0	0	Healthy subjects no history of knee pain or trauma	Simulated squat within scanner	200 N (40° knee flexion)	NR	Thickness	3.0T	1	Patellar
Marsh et al. [53]	25 Healthy: 8 OA-KL2: 9 OA-KL3: 8	55 (7)	28.0 (2.2)	100	Females > 40 yrs Healthy: no history pain/stiffness most days past year/KL0 OA: history pain/stiff most days past month/KL2 or 3	Simulated stand within scanner	50% BW (15° knee flexion)	10	Thickness	3.0T	1	Medial tibiofemoral
Mayerhoefer et al. [68]	10	27 (5)	NR	30	Asymptomatic volunteers, no history knee injury/surgery/focal MRI cartilage defects	Simulated stand within scanner	50% BW	NR	T1 dGEMRIC	3.0T	6	Tibial Femoral
Nag et al. [69]	26	52 (15)	NR	46	Asymptomatic subjects, no knee pain/symptoms last 3 yrs	Simulated single leg stand within scanner	60 kg	20	T2 relaxation time	1.5T	12	Tibial Femoral
Niehoff et al. [54]	14	23 (2)	22.5 (1.8)	50	Healthy sedentary young adults (20–30 yrs; BMI 20–30 kg/m^2^), no history lower limb pain/symptoms/injury	Double leg drop jump	100 reps (from 73 cm height)	30	Thickness, volume	1.0T	5	Patellar Tibial Femoral
Nishii et al. [55]	22	25 [21–43]	21.4 [18.0–29.7]	59	No history knee pain/stiffness/surgery	Simulated stand within scanner	50% BW	21	Thickness, T2 relaxation time	3.0T	12	Tibial Femoral
Nishii et al. [75]	24 Healthy: 9 Hip dysplasia:15	Healthy: 28 [23–40] Hip dysplasia: 39 [24–50]	Healthy: 20.8 [18.9–23.2] Hip dysplasia: 21.9 [18.9–29.5]	100	Women Healthy: no hip pain/stiffness/limited ROM/gait disability, Hip dysplasia: CEA ≤ 24°, no surgery, class I subluxation, KL0-2	Simulated stand within scanner	50% BW	19.5	Thickness, T2 relaxation time	3.0T	12	Weightbearing lateral hip
Owusu-Akyaw et al. [56]	8 (16 knees) Healthy knees: 8 ACLD knees: 8	31 [21–47]	25.6 [21.7–34.7]	0	Male subjects with unilateral ACLD Healthy knee: clinically & radiologically intact ACL, no history injury ACLD knee: ACL injury, no reconstruction/other surgery	Hop	60 reps (0.6 m horizontal distance)	NR	Thickness	3.0T	1	Patellar
Paranjape et al. [57]	10	25 [22–27]	22.1 [20.0–24.7]	50	Healthy young adults, no history lower limb pain/symptoms/injury	Walk	Fr = 0.25 (mean 5.47 km/hr)	30	Thickness	3.0T	3	Tibial
Schoenbauer et al. [70]	9	31 (7)	NR	33	Healthy volunteers, no history knee pain/stiffness/surgery	Simulated stand within scanner	125 N	48	T2 relaxation time	3.0T	4	Tibial Femoral
Schütz et al. [58]^c^	4 (8 knees)	33.0	NR	0	Experienced alpine skiers with no relevant abnormalities on clinical or MRI examination	Alpine skiing	Category blue/red slopes	1 h With/without damped ski binding	Thickness T2* relaxation time	1.5T	5	Patellar Tibial Femoral
Sitoci et al. [59]	17 Sub-group: 8	F: 22 (3) M: 24 (3)	F: 22.2 (2.2) M: 21.5 (2.1)	47	Healthy young subjects, no history knee symptoms/signs/trauma/surgery	Evening: Knee bends Single leg sustained squat	30 reps (120° knee flexion) Sub-group: 1 rep (15° knee flexion) (150% BW)	1.5 Sub-group: 3.5	Thickness	1.5T	1 Sub-group: 3	Patellar Sub-group: Tibial
Souza et al. [71]^c^	30 Healthy KL0: 10 OA-KL2/3: 20	Healthy: WORMS = 0–53 (7) OA: WORMS > 0–57 (5)	Healthy: WORMS = 0–27.9 (2.6) OA: WORMS > 0–28.0 (2.0)	100	Women > 40 yrs, BMI 25–35 Healthy: KL0, no history knee pain/surgery/disease OA: KL2 or 3, history pain/stiffness most days past month, knee with most severe OA scanned	Simulated stand within scanner	50% BW (20°knee flexion)	> 20	T2 relaxation time, T1ρ relaxation time	3.0T	2	Medial & lateral Tibiaofemoral
Souza et al. [72]	137 Healthy: 93 OA: 44	Healthy: 50 (95% CI 48–51) OA: 57 (95% CI 54–60)	Healthy: 24.0 (95% CI 23.3–24.7) OA: 26.4 (95% CI 24.0–28.7)	Healthy: 58 OA: 61	Adults > 35 yrs, no history knee fracture/surgery Healthy: KL ≤ 1, no history knee pain/stiffness/medication in past 1 year OA: KL > 1, history pain/stiffness/medication use most days of month in past year	Simulated stand within scanner	50% BW (20° knee flexion)	43	T2 relaxation time, T1ρ relaxation time	3.0T	14	Tibial Femoral
Stehling et al. [74]	30 Healthy KL0: 10 OA-KL2/3: 20	Healthy WORMS = 0: 56 (NR) OA WORMS > 0: 56 (NR)	Healthy WORMS = 0: 28 (NR) OA WORMS > 0: 27 (NR)	100	Women > 40 yrs, BMI 25–35 Healthy: KL0, no history knee pain/surgery/disease OA: KL2 or 3, history pain/stiffness most days past month, knee with most severe OA scanned	Simulated stand within scanner	50% BW (20° knee flexion)	20	Semi-quantitative (WORMS)	3.0T	6	Patellar Tibial Femoral
Subburaj et al. [73]	30 Healthy KL0: 10 OA-KL2/3: 20	Healthy WORMS = 0: 56 (NR) OA WORMS > 0: 57 (NR)	Healthy WORMS = 0: 28 (NR) OA WORMS > 0: 27 (NR)	100	Women 40–70 yrs, BMI 20–35 Healthy: KL0, no history knee pain/surgery/disease OA: KL2 or 3, history pain/stiff most days past month, knee with most severe OA scanned	Simulated standing within scanner	50% BW (20° knee flexion)	20	Thickness	3.0T	4	Tibial Femoral
Sutter et al. [61]^3^	8 (16 knees)	26 [24–30]	22.8 [21.1–25.1]	0	Males with no history of injury or surgery to either knee	Hop	60 reps (0.6 m horizontal distance)	NR	Thickness	3.0T	42	Tibial Femoral
Sutter et al. [60]	8 (16 knees) Healthy knees: 8 ACLD knees: 8	31 [21–47]	25.6 [21.7–34.7]	0	Males Healthy: intact contralateral knee, no history injury/surgery ACLD: ACL deficient knee	Hop	60 reps (0.6 m horizontal distance)	NR	Thickness	3.0T	5	Tibial Femoral
Tamayo et al. [62]^c^	15 Healthy BMI: 8 High BMI: 7	Healthy BMI: 30 [22–45] High BMI: 32 [22–45]	Healthy BMI: 22.8 [18–55] High BMI: 32.8 [30–36]	Healthy MI: 38 High BMI: 57	No history lower limb injury/surgery/OA symptoms	Walk	Fr = 0.25 (mean 5.47 km/hr)	20	Thickness T1ρ relaxation time	3.0T	1	Patellofemoral
Van Ginckel et al. [63]	30 Healthy: 18 OA-KL1/2: 18	Healthy: median 43 [IQR:5] OA-KL1/2: median 55 [IQR: 14]	Healthy: 24.0 (3.5) OA-KL1/2: 27.1 (3.7)	Healthy: 33 OA-KL1/2: 33	Adults 40–60 yrs Healthy: no history knee pain/injury/surgery OA-KL1 or 2, WORMS ≥ 2, no history knee cartilage surgery or arthroplasty/hyaluronan injection in past 3 months/other joint pathology	Knee bends	30 reps (90° knee flexion)	1	Volume	3.0T	4	Tibial Femoral
Verschueren et al. [31]	10	24 (2)	23.5 (2.9)	50	Healthy volunteers	Stationary cycle	Intermediate pace	10	T2 relaxation time	3.0T	3	Tibial Femoral
Wang et al. [64]	4	35 (10)	NR	75	Healthy volunteers	Immediate (a) Simulated standing within scanner	50% BW (0° knee flexion)	10	Thickness	3.0T	4	Tibial Femoral

*ACLD* anterior cruciate ligament deficient, *ACLR* anterior cruciate ligament reconstruction, *BMI* body mass index calculated from height and weight (kg/m^2^), *BW* body weight, *CEA* centre-edge angle, *CI* confidence interval, *cm* Centimetre, *Fr* Froude number (calculated from walk speed [*velocity*, in m/s], lower limb length [m, floor to the greater trochanter], and the gravitational constant [9.81, in m/second^2^] and Fr = 0.25 [~ 1.48 ± 0.05 m/s] represents a comfortable walking pace for adults), *F* female, *Gp* group, *IQR* interquartile range, *KL* Kellgren–Lawrence OA grade (0–4), km/hr = kilometres per hour, *M* male, *m* metres, *min* minutes, *N* newtons, *NR* not reported, *OA* osteoarthritis, *PFJ* patellofemoral joint cartilage, *PFP* patellofemoral pain, *ROM* range of motion, *reps* repetitions, *rpm* revolutions per minute, *WB* weightbearing, *WOMAC* Western Ontario and McMaster Universities index for knee OA, *SD* standard deviation, *TFJ* tibiofemoral joint cartilage, *WORMS* Whole-Organ MRI Score; *yrs* years

^a^Data obtained from author

^b^Study may have reported other data that were not eligible for inclusion in review meta-analyses

^c^Bilateral, incomplete or cartilage region data ineligible for meta-analysis

Knee cartilage thickness and/or volume changes were measured in 28 studies (25 and 6, respectively) [19, 39–64] and compositional changes in 16 studies (T2 = 12, T1ρ = 7, T2* = 1 and dGEMRIC = 1) [31, 46, 47, 50, 55, 58, 62, 65–73]. One study used a semi-quantitative measure of knee cartilage defects [74]. Hip cartilage was investigated using MRI thickness and T2 [75] and T2* relaxation time [76] measures. Online software was used to obtain percentage change or pre- and post-activity measures from graphed results, when authors were unable to supply the requested data [39, 41–46, 53, 54, 56, 57, 59–61, 64, 66–68, 76].

Joint loading activity included sustained compression loading (50% body weight) applied to the foot of individuals lying within the scanner to simulate upright standing (mean duration 24 ± 13 min) [40, 50, 53, 55, 64, 68–73, 75] and a knee flexed protocol was used to simulate squatting [52, 67]. Included studies also investigated walking [39, 41–43, 48, 51, 57, 62, 65, 66] (mean duration 21 ± 9.7 min) and cycling [31, 43, 47] (mean duration 22 ± 9 min). Hopping [56, 60], double-leg [54] and single-leg drop-jumps [43] were considered similar (hop/jump) activities and combined in meta-analyses. One study investigated the effects of skiing for 1 h [58]. Nine studies investigated the impact of bilateral, body weight loaded knee flexion (90–120°) activities (described as knee-bends), repeated (mean ± SD) 38 ± 10 times for 1–2 min [19, 43–46, 49, 59, 63, 76]. Two studies measured the effects of an activity at different intensities (on separate occasions) [45, 57]. To optimise analysis homogeneity, we selected cartilage change data obtained after 30 min walking from a study investigating separate walking bouts of different durations (10, 20, 30, 40, 60 min) with normalised speed [57] and after 50 repeated knee bends (in preference to 100 repetitions) [45].

### Risk of Bias and Certainty of Evidence

**Fig. 2:**
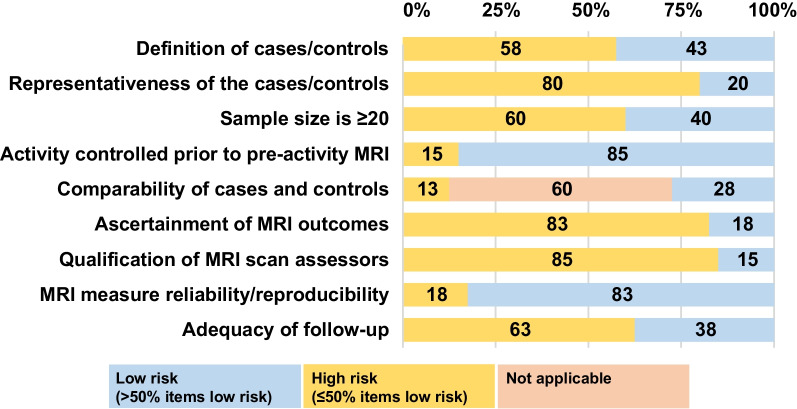
Summary of risk of bias using the Newcastle–Ottawa Scale

Nine of 40 studies (23%) were assessed as low risk of bias (Fig. 2, Additional file 3). Most studies (85%) controlled participant activity prior to pre-exercise MRI [19, 39–48, 51–53, 56–66, 68–76] and 86% reported reproducibility and/or reliability of their MRI techniques. However, few studies (17%) were assessed as low risk of all (three) participant selection bias items [40, 46, 63, 71, 72, 74], blinded MRI readers (19%) [19, 40, 48, 54, 65, 68, 71] or reported MRI reader qualifications and reliability (15%) [19, 39, 50, 52, 62, 63, 75, 76]. High risk of bias, together with other components of the GRADE criteria, indicated very low certainty of evidence for all findings (Additional file 4).

### Immediate Knee Cartilage Thickness and Volume Changes in Healthy Adults

**Fig. 3:**
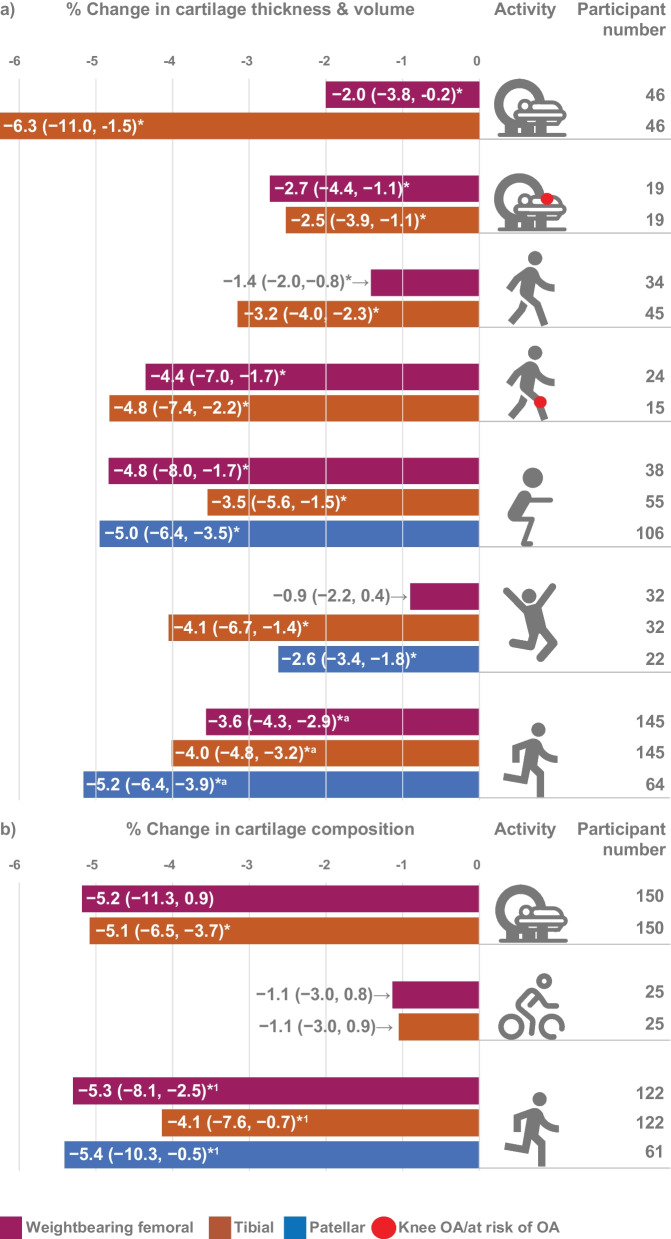
Summary of MRI cartilage **a** thickness and volume and **b** composition percent change after activity. Summary includes analyses with two or more datasets, *Percent change significant *p* < 0.05, ^1^Run pooled meta-analysis sourced from previously published systematic review [10]. MRI icon from Eucalyp, www.flaticon.com

**Fig. 4:**
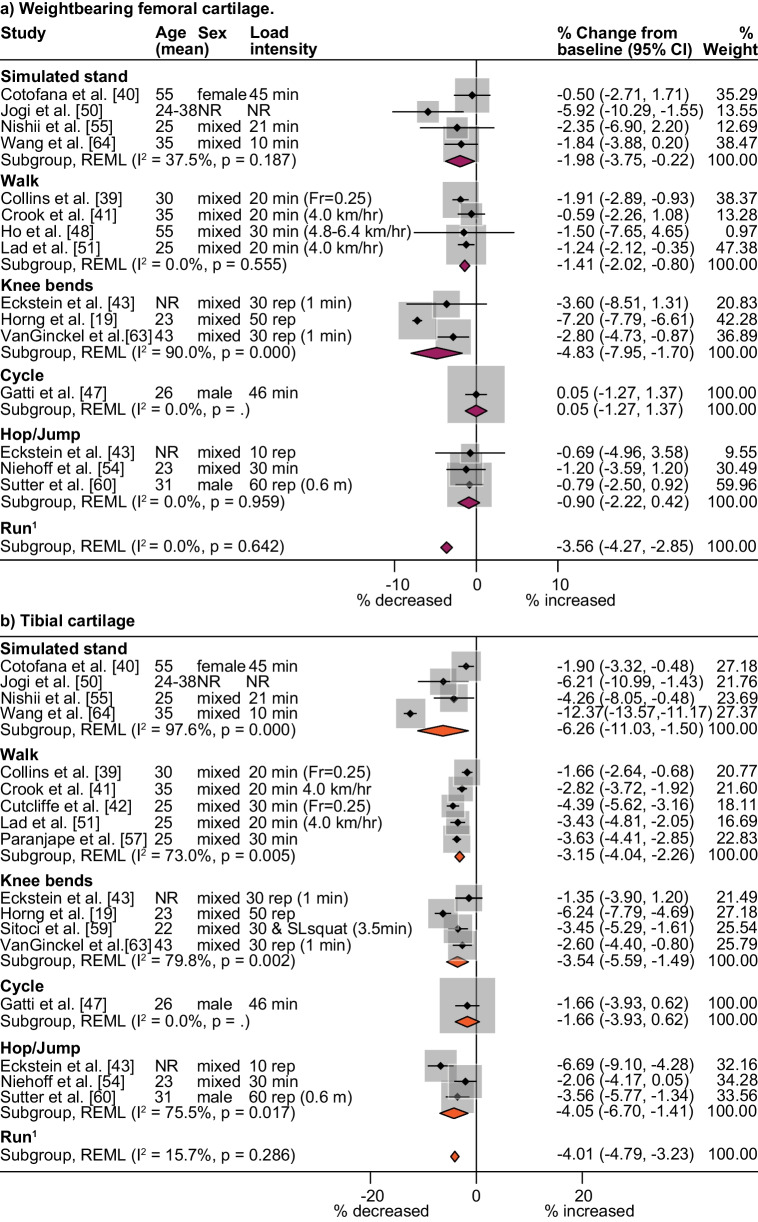
Percent change in tibiofemoral cartilage thickness and volume after different activities. ^1^Run pooled meta-analysis sourced from previously published systematic review [10], *CI* Confidence interval, *Fr* Froude number (calculated from walking velocity [m/s], lower limb length [m, floor to the greater trochanter], and the gravitational constant [9.81, in m/s^2^] and Fr = 0.25 (1.48 ± 0.05 m/s) represents comfortable walking pace for adults, *hr* hour, *km* kilometres, *m* metres, *min* minutes, *NR* not reported, *rep* repetitions, *s* seconds, *SL* single leg

**Fig. 5:**
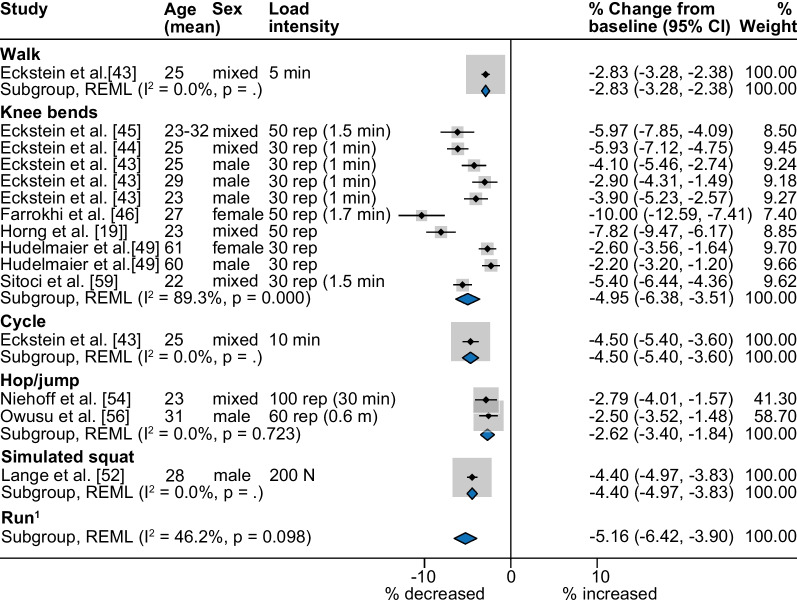
Percent change in patellar cartilage thickness and volume after different activities. ^1^Run pooled meta-analysis sourced from previously published systematic review [10], *CI* confidence interval, *m* metres, *min* minutes, *N* newtons, *rep* repetitions

Knee cartilage thickness and volume decreased immediately after most joint loading activities (Fig. 3), but weightbearing femoral cartilage changes were not significant after hopping/jumping and reductions were minimal after walking (2.0%, 95% CI 0.2–3.8%, *I*^2^ = 37.5%) (Fig. 4a). Reductions in tibial cartilage thickness and volume were smallest after walking (3.2%, 95% CI 2.3–4.0%, *I*^2^ = 73.0%) and greatest after simulated standing (6.3%, 95% CI 1.5–11.0%, *I*^2^ = 97.6%) (Fig. 4b). The largest immediate reductions in patellar cartilage thickness and volume occurred following knee bends (5.0%, 95% CI 3.5–6.4%; *I*^2^ = 89.3%) (Fig. 5). Single studies indicated that cycling at 80 Hz for 10 min on a stationary bike produced a 4.5% reduction in patellar cartilage (95% CI 3.6–5.4%) (Fig. 5) [43], yet cycling at the same rate for 45 min resulted in no significant change in weightbearing femoral or tibial cartilage thickness and volume. The only study to semi-quantitatively measure knee cartilage lesions found that WORMS cartilage score, signal intensity or lesion shape did not change in healthy participants after simulated standing [74].

### Immediate Knee Cartilage Composition Changes in Healthy Adults

**Fig. 6:**
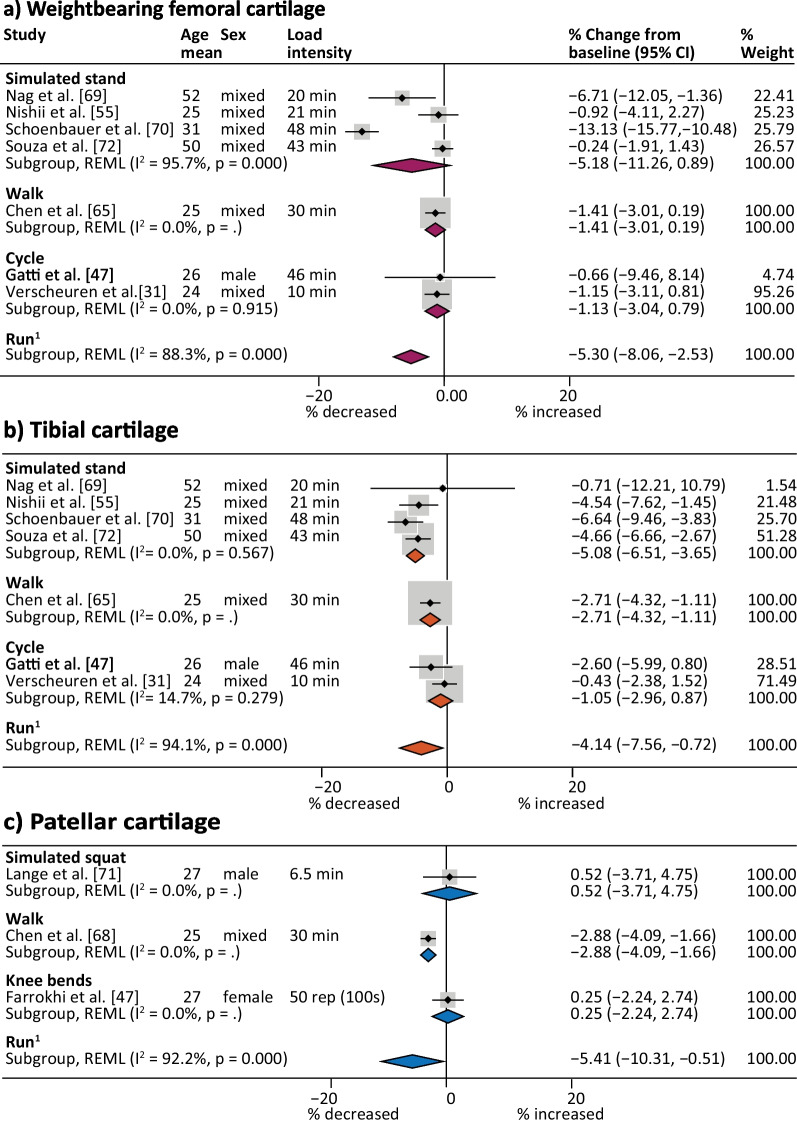
Percent change in cartilage composition after different activities. ^1^Run pooled meta-analysis sourced from previously published systematic review [10], *CI* confidence interval, *min* minutes, *NR* not reported, *rep* repetitions, *s* seconds

Simulated standing within the scanner resulted in highly variable and not statistically significant reductions in weightbearing femoral cartilage T2 relaxation time (5.2%, 95% CI − 0.9–11.3, *I*^2^ = 95.7%) but reduced tibial T2 relaxation times by 5.1% (95% CI 3.7–6.5%, *I*^2^ = 0%) (Figs. 3 and 6). Cycling did not appear to impact weightbearing femoral or tibial cartilage relaxation times (Fig. 6). A single study found that a 30 min walk reduced T2 relaxation time 2.7% (95% CI 1.1–4.3%) in tibial cartilage but had no effect on weightbearing femoral cartilage. Only single studies evaluated compositional changes in patellar cartilage after activity, with walking resulting in a 2.9% (95% CI 1.7–4.1%) decrease in T2 relaxation time [65] and no significant changes after simulated squat within the scanner [67] or after knee bends [46] (Fig. 6c). Incomparable cartilage regions and activities precluded pooling of data from studies that evaluated T1ρ outcomes [62, 65–67, 71, 72, 77], but individual studies reported reductions in tibial cartilage that ranged from 3.2% (± 2.8%) after walking [65] to 8.2% (± 10.6%) after simulated standing [72]. T1-dGEMRIC relaxation time decreased significantly in weightbearing femoral and tibial cartilage (6.6%, ± 10.5% and 6.1%, ± 9.2%, respectively) after simulated standing [68].

### Delayed Knee Cartilage Changes in Healthy Adults and Those with OA

Four studies investigated cartilage recovery after different activities with repeated, delayed MRI (20 min–48 h) post-activity [31, 42, 45, 63]. Loss of tibial cartilage thickness and volume had recovered at 25.2 min (root mean square error = 0.24, line fit = 0.46) after a 30 min walk in healthy adults [42]. Significant tibiofemoral cartilage reductions measured immediately after 30 knee bends in healthy individuals and those with mild OA had recovered to baseline within 15 min in both groups [63]. Patellar cartilage has been reported to recover in an approximately linear pattern following 100 knee bends, with approximately 50% recovery of thickness and volume at 45 min and near complete recovery at 90 min in healthy adults [45]. T2 relaxation time recovered in superficial cartilage subregions after simulated standing [70]. However, a small increase in T2 relaxation time measured in the lateral tibial plateau following 10 min cycling in healthy adults had not recovered 20 min later and had also increased in the lateral femoral cartilage [31].

### Immediate Knee Cartilage Changes in Adults with, or at Risk of, OA

Eleven studies evaluated immediate cartilage thickness and volume changes [39–41, 46, 48, 53, 56, 60, 62, 63, 73] and six studies evaluated composition changes [46, 62, 71, 72, 78, 79] in individuals with, or at risk of, knee OA. Comparability of cartilage regions and activities was limited; two pooled analyses identified reductions in weightbearing femoral and tibial cartilage thickness and volume following simulated standing (2.7% and 2.5%, respectively) and walking (4.4% and 4.8%), respectively (Additional files 5 and 6). A single study of individuals with patellofemoral pain found smaller patellar cartilage thickness and volume changes (4.4%, ± 3.3%) after 50 knee bends, compared to healthy participants (10.0%, ± 4.2%), but no composition differences [46]. Similarly, smaller reductions in patellar cartilage thickness (2.5%, ± 1.4) have been reported in ACL deficient knees compared to the intact contralateral knees (5.4%, ± 1.1) after 60 hops [56]. In contrast, tibiofemoral cartilage volume reductions after 30 knee bends were found to be similar in individuals with mild OA (Kellgren–Lawrence [KL] = 1–2) and healthy participants [63]. Subregion analysis of the medial femoral condylar cartilage, immediately adjacent to the intercondylar notch, decreased more in ACL deficient knees than healthy contralateral knees after hopping [60]. The only study to semi-quantitatively measure changes in knee cartilage lesions found that 16.6% of participants with radiographic OA (KL = 2–3) had increased WORMS cartilage score, signal intensity or lesion shape after simulated standing [74]. Greater reductions in tibiofemoral [39] and patellofemoral [62] cartilage thickness after (20 min) walking were found in individuals with high BMI compared to participants with normal BMI.

### Immediate Hip Cartilage Changes in Healthy Adults and Those at Risk of OA

From the two studies that evaluated immediate changes in hip cartilage after activity, thickness and volume did not change after simulated standing but individuals with hip dysplasia had significant post-activity reductions in peripheral acetabular cartilage thickness (7.9%, ± 11.5%) and T2 relaxation time (7.6%, ± 10.6%) [75]. The second study investigated hip cartilage composition (using T2* relaxation time) following 50 knee bends in healthy adults and found no changes compared to pre-activity [76].

### The Effects of Sex, Age, and Activity Duration/Repetitions

**Fig. 7:**
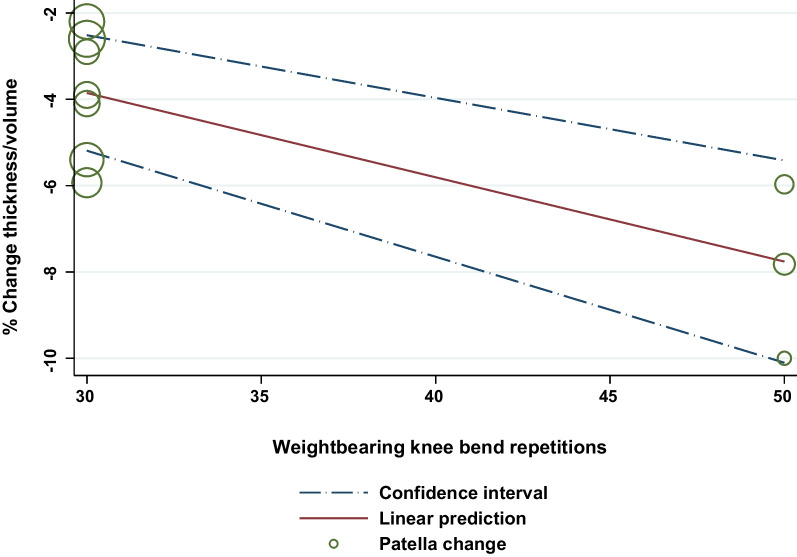
Patellar cartilage thickness and volume changes associated with the number of knee bends

There were generally no other associations between knee cartilage thickness and volume changes and sex, age or joint loading activity duration (Additional file 7). However, for repeated weightbearing knee bends, for every increase of 10 repetitions, patellar cartilage thickness and volume reduced by 2% (95% CI 0.6–3.3%) (Fig. 7).

## Discussion

Our systematic review is the first to investigate immediate and delayed changes to knee and hip cartilage after joint loading activities other than running. The results build on our recent systematic review (immediate effects of running) from the same overarching protocol [10]. Broadly, knee cartilage thickness, volume and composition reduced immediately following various activities, but changes were small (0–6%) with the largest immediate reductions in thickness and volume and composition after simulated standing. MRI-assessed cartilage changes after joint loading activities could be explained by the current multiphasic models of normal cartilage viscoelastic response to loading, which describe the redistribution and exudation of small amounts of water [80, 81]. The current data, and our previous findings, support MRI research protocols that utilise a period of 30 min of non-weightbearing prior to quantifying cartilage [18] and avoiding strenuous activity (i.e. repeated squatting, running) for at least 90 min to 48 h prior to the scan. Our findings were graded very low certainty due to limitations associated with high risk of bias and heterogeneity of included studies. Only two of the forty eligible studies investigated hip cartilage changes after activity, which may reflect the challenges associated with obtaining reproducible and sensitive MRI measures of hip cartilage [82].

We found the magnitude of change depended on the cartilage region and activity assessed. For example, 1–2 min of repeated knee bends (e.g. 30–50 repetitions, knee flexion 90–110°) appeared to have a greater impact on weightbearing femoral (− 4.8%) and a similar impact on patellar (− 5.0%) cartilage thickness and volume as running (− 3.6% and 5.2%, respectively) for (mean) 33 min [10]. This result is perhaps surprising given the shorter overall activity duration of repeated knee bends and high magnitude forces estimated to be five times body weight when running [83]. The cartilage effects of repeated knee bends could be due to activity parameters such as longer instances of load (1–2 s repetitions) which were up to tenfold longer than the 0.2–0.3 s load time estimated to occur during each stance phase of running [84]. Weightbearing femoral cartilage contact with the patella can occur during the maximum compressive force of a knee bend at 90° flexion [85] which has been estimated to be high (up to 18 times body weight) and associated with activities requiring greater knee flexion [43, 83]. We also identified a dose–response to loading in patellar cartilage where every increase of 10 knee bend repetitions resulted in decreases in cartilage thickness and volume by 2%. This finding aligns with larger reductions in patellar and tibial cartilage thickness and volume following increased run [86] and walk [57] durations, with controlled gait speed. Delayed (21 min–48 h) cartilage changes could not be pooled due to insufficient data, but individual studies indicated that cartilage reductions in tibial and femoral thickness and volume recovered within 15 min after knee bends [63]. Partial recovery of patellar cartilage volume occurred between repeated bouts of (50) knee bends spaced 15 min apart [45], but 90 min was required to completely recover after one bout of 100 knee bends [45]. As weightbearing knee bends, or “squats”, are a common component of rehabilitation and fitness programs, our findings could guide program design by incorporating bouts of fewer, faster knee bend repetitions to minimise patellofemoral cartilage effects.

Static unilateral application of 50% body weight loading within the scanner (to simulate upright standing) resulted in 20% greater tibial composition reduction than we found in tibial cartilage after running [10]. Reduced cartilage compositional measures (e.g. T2 relaxation times) have been associated with reduced free water [87], a more consolidated cartilage collagen matrix [88] and are related to normal viscoelastic behaviour of loaded cartilage. The cartilage effects of simulated standing could also illustrate the time-dependent behaviour of healthy cartilage, as sustained body weight loading appears to have a similar impact on composition as cyclic instances of brief but higher magnitude loads of running. It is also possible that scans taken during simulated standing better reflect the immediate effects of loading, as some recovery of cartilage composition may have already occurred in the running studies, in which scans were commenced (minutes) after activity completion [10]. Static unilateral application of 50% body weight loading within the scanner may not actually simulate standing in vivo as the contribution of weight-shifts and minor load perturbations that likely maintain cartilage thickness and volume during upright standing [89] have been eliminated by more passive supine positioning combined with foot and trunk fixation to minimise movement within the scanner. We found smaller composition reductions in OA populations, which may reflect heterogeneity in the severity of OA disease, as early stages of disease are more responsive to therapeutic exercise load [8].

Walking is an important mode of physical activity for older adults, with the accumulation of at least 10,000 steps per day thought to lower risk of mortality [90]. We found reductions in weightbearing femoral and tibial cartilage thickness and volume after walking for a (mean) duration of 21 min were smaller (1–3%) than with all other activities. Patellar cartilage thickness and volume reductions after walking (3%) (measured in only one study) were approximately half the magnitude measured after running [10], which aligns with smaller patellofemoral joint reaction forces measured during walking [83]. Greater tibiofemoral cartilage thickness and volume changes may occur at higher walking speeds [57]; however, there were insufficient data to pool the cartilage effects of different walking load intensities. Our analyses found that individuals with, or at risk of, OA, appear to have a larger reduction in tibial and weightbearing femoral cartilage thickness and volume compared to healthy individuals. Due to limited data, we were unable to compare responses of individuals with OA and those at risk of OA who may be at different stages on the degeneration continuum. Current evidence, limited to only one study, indicates a gradual, nonlinear recovery of healthy tibial cartilage thickness and volume after walking 30 min, with complete recovery occurring after 25 min [42]. The transient nature of these immediate cartilage thickness and volume changes is consistent with those observed after running where tibiofemoral cartilage returns to baseline levels within 60–90 min post-run [10], suggesting that (at least a single bout of) walking (and running) is not detrimental to knee joint health.

From limited data, narrative synthesis indicates that hip cartilage thickness and volume reductions are smaller than those observed in the knee for the same activities (i.e. simulated standing, knee bends). However, for individuals at risk of hip OA (i.e. those with hip dysplasia), simulated standing appears to result in significant loss of acetabular cartilage thickness and quality. Hip cartilage may be less responsive to load compared to knee cartilage and contribute to occupational loads being less of a factor in the development of OA in the hip compared to the knee [91]. However, further research evaluating the impact on different activities on hip cartilage and OA is needed.

### Limitations

Building on recent systematic reviews of the immediate effects of running on lower-limb cartilage, we included data from all other joint loading activities in the current review. However, there were limited data evaluating tasks such as hopping, jumping, step down and cycling, with generally small changes in thickness and volume and composition observed. A single study did find a large reduction in patellar cartilage morphology after cycling, similar to running and repeated knee bends, potentially due to the increases in patellofemoral joint forces with cycling intensity [92, 93]. As a relatively new tool, MRI evaluation of articular cartilage is expensive and time intensive, and so the number of participants in included studies was small (mean *n* = 17). The number of studies in each meta-analysis was small due to the variety of activities investigated, cartilage regions reported, and MRI measures used and limited studies measuring individuals with, or at risk of, OA. Averaging sub-region MRI outcome measures may have diluted smaller and larger changes in cartilage after activity, although small sub-region analyses are thought to be less reliable due to high heterogeneity [94]. Caution should be adopted when making comparisons across studies in this review due to the variability in MRI sequences and equipment used that may have confounded the pooling of results [95]. This supports the need to address standardisation of MRI methodology [96] to improve certainty in the body of evidence, which was very low in our GRADE evaluation. Nevertheless, a key strength of most of the included studies was the standardisation of a period of non-weightbearing prior to the first MRI scan acquisition. This is a recommendation for MRI reproducibility [18, 29], particularly for all compositional sequences, which can be confounded by pre-scan joint loading, as confirmed in our review.

## Conclusion

We found very low certainty evidence for small percent changes in knee cartilage thickness and volume and composition (0–5%) following all activities investigated. This is the first review to synthesise the evidence regarding the effects of everyday joint loading activities and rehabilitation-type exercises on knee and hip cartilage, using MRI measures. There are minimal data about the effect of joint loading activities on hip cartilage. From limited data available, it appears that most of these immediate changes were transient in healthy adults, suggesting that bouts of walking, cycling, squatting and jumping do not adversely impact cartilage health in the short term. While we know less about those with OA, our findings could be useful for clinicians when designing and prescribing rehabilitation programs and providing exercise advice. Based on current evidence, patients with knee OA should be educated that the benefits of these activities (i.e. physical, mental health and well-being) are likely to outweigh the risks for cartilage health.

## Supplementary Information

**Additional file 1:** Search strategy.

**Additional file 2:** The modified Newcastle-Ottawa Quality Assessment Scale (NOS) for risk of bias assessment.

**Additional file 3:** Risk of bias assessment using the NOS.

**Additional file 4:** GRADE Certainty of the evidence assessment.

**Additional file 5:** Percent change in tibiofemoral cartilage thickness and volume in participants with, or at risk of, OA.

**Additional file 6:** Percent change in tibiofemoral cartilage composition in participants with, or at risk of, OA.

**Additional file 7:** Summary of meta-regression analyses exploring associations between sex, age, and activity duration/repetitions and cartilage thickness and volume changes.

## Data Availability

The datasets generated and/or analysed during the current study are available from the corresponding author on request.

## Abbreviations

ACL: Anterior cruciate ligament
BMI: Body mass index
CI: Confidence interval
dGEMRIC: Delayed Gadolinium-enhanced MRI of cartilage
GRADE: Grading of Recommendations Assessment, Development and Evaluation
*I*^2^: Variation across studies due to heterogeneity
*K*: Kappa
KL: Kellgren–Lawrence OA severity classification
MRI: Magnetic resonance imaging
NOS: Newcastle–Ottawa Scale
OA: Osteoarthritis
PRISMA: Preferred Reporting Items for Systematic Reviews and Meta-Analyses
REML: Restricted maximum likelihood
SHOMRI: Scoring Hip Osteoarthritis with MRI
SD: Standard deviation
WORMS: Whole-Organ MRI Score
