# Supplementary material for: Immediate and Delayed Effects of Joint Loading Activities on Knee and Hip Cartilage: A Systematic Review and Meta-analysis
Source: Sports Med Open. 2023 Jul 14;9:56. doi: 10.1186/s40798-023-00602-7 (PMC10348990; doi:10.1186/s40798-023-00602-7)
Supplement: Supplementary file 1 — Additional file 1: Search strategy. [file 40798_2023_602_MOESM1_ESM.docx]

**Online Resource 1. Search strategy.**

Original search strategies were devised for this review.

Each database was searched separately on each platform using customised methods and terms described below.

The first search for all databases occurred on 6 July, 2020.

The search was updated in June 2021 and April, 2023.

Search updates were performed by rerunning all saved database search strategies within specified dates.

MEDLINE (via Ovid)

1. Knee/ or Knee joint/ or Patella/ or Patellofemoral joint/

2. Hip Joint/ or Hip/ or Groin/

3. Anterior cruciate ligament/ or Posterior cruciate ligament/ or Medial collateral

ligament/ or Menisci, tibial/

4. Osteoarthritis, Knee/ or Knee injuries/ or Patellofemoral pain syndrome/ or Anterior cruciate ligament injuries/ or Tibial meniscus injuries/ or Knee dislocation/ or Patella dislocation/ or Chondromalacia patellae/ or Osteoarthritis, hip/ or Hip injuries/ or Femoroacetabular impingement

5. knee* or patell* or PFP or PFPS or AKP or tibial or tibiofemoral or menisc* or anterior cruciate ligament* or ACL or posterior cruciate ligament* or PCL or ((medial or lateral) adj3 ligament*) or MCL or LCL).tw,kf.

6. (hip* or femor* or FAI or FAIS or acetabular or groin*).tw,kf.

7. or/1-6

8. Exercise/ or Exercise therapy/ or Exercise movement techniques/ or Physical exertion/ or Physical fitness/ or Physical endurance/ or Muscle strength/

9. Rehabilitation/ or Resistance training/ or Endurance training/ or Plyometric exercise/

10. exp Sports/

11. Weight-bearing/ or exp Gait/

12. (exercis* or rehabilitat* or (muscle* adj3 strength*) or closed kinetic chain or open

kinetic chain).tw,kf.

13. ((isotonic* or isokinetic* or isometric* or plyometric* or strength* or resist* or weight* or endurance or aerobic) adj3 (train* or exercis*)).tw,kf.

14. (run* or jog* or walk* or gait* or bicycl* or cycl* or treadmill or marathon or physical activit*).tw,kf.

15. (weight-bear* or weightbear* or (joint* adj3 (load* or force*))).tw,kf.

16. or/8-15

17. Magnetic Resonance Imaging/

18. ((magnetic resonance adj3 imag*) or MR or MRI or T1* or T2* or dGEMRIC).tw,kf.

19. 17 or 18

20. Cartilage/ or Articular cartilage/ or Collagen/ or Glycosaminoglycan/

21. (cartilag* or chondr* or collagen* or glycosaminoglycan*).tw,kf.

22. (SHOMRI or WORMS or MOAKS or ACLOAS or BLOKS or KOSS).tw,kf.

23. or/20-22

24. 7 and 16 and 19 and 23

EMBASE (via Ovid)

1. Knee/ or Patella/ or Patellofemoral joint/

2. Hip/

3. Anterior cruciate ligament/ or Posterior crcuiate ligament/ or Knee medial collateral ligament/ or Knee meniscus/

4. Knee osteoarthritis/ or Knee injury/ or Patellofemoral pain syndrome/ or Patella chonromalacia/ or Anterior cruciate ligament injury/ or Posterior cruciate ligament injury/ or knee ligament injury/ or Tibial meniscus injuries/ or Knee dislocation/ or Patella dislocation/

5. Hip osteoarthritis/ or Hip injury/ or Femoroacetabular impingement/ or Inguinal pain/

6. knee* or patell* or PFP or PFPS or AKP or tibial or tibiofemoral or menisc* or anterior cruciate ligament* or ACL or posterior cruciate ligament* or PCL or ((medial or lateral) adj3 ligament*) or MCL or LCL).tw,kw.

7. (hip* or femor* or FAI or FAIS or acetabular or groin*).tw,kw.

8. or/1-7

9. Exercise/ or Kinesiotherapy/ or Muscle strength/

10. Muscle training/ or Functional training/ or Athletic rehabilitation/

11. exp Sport/

12. Physical activity/ or Gait/

13. (exercis* or rehabilitat* or (muscle* adj3 strength*) or closed kinetic chain or open

kinetic chain).tw,kw.

14. ((isotonic* or isokinetic* or isometric* or plyometric* or strength* or resist* or weight* or endurance or aerobic) adj3 (train* or exercis*)).tw,kw.

15. (run* or jog* or walk* or gait* or bicycl* or treadmill or marathon or physical activit*).tw,kw.

16. (weight-bear* or weightbear* or (joint* adj3 (load* or force*))).tw,kw.

17. or/9-16

18. Nuclear Magnetic Resonance Imaging/

19. ((magnetic resonance adj3 imag*) or MR or MRI or T1* or T2* or dGEMRIC).tw,kf.

20. 18 or 19

21. Cartilage/ or Articular cartilage/ or Collagen/ or Glycosaminoglycan/

22. (cartilag* or chondr* or collagen* or glycosaminoglycan*).tw,kf.

23. (SHOMRI or WORMS or MOAKS or ACLOAS or BLOKS or KOSS).tw,kf.

24. or/21-23

24. 8 and 17 and 20 and 24

CINAHL (via EBSCOhost)

1. (MH "Knee") OR (MH "Knee Joint+") OR (MH "Patella") OR (MH "Hip") OR (MH "Hip Joint")

2. (MH "Anterior Cruciate Ligament") OR (MH "Posterior Cruciate Ligament") OR (MH "Medial Collateral Ligament, Knee") OR (MH "Menisci, Tibial")

3. (MH "Knee Injuries") OR (MH "Knee Dislocation+") OR (MH "Knee Injuries, Articular Cartilage") OR (MH "Osteoarthritis, Knee") OR (MH "Anterior Cruciate Ligament Injuries") OR (MH "Posterior Cruciate Ligament Injuries") OR (MH "Medial Collateral Ligament Sprain") OR (MH "Meniscal Injuries") OR (MH “Chondromalacia Patella”) OR (MH "Patellofemoral Pain Syndrome") OR (MH “patella dislocation”)

4. (MH “Hip injuries” OR (MH “Hip Labrum Tear”) OR (MH "Femoracetabular Impingement") OR (MH "Groin Pain") OR (MH "Osteoarthritis, Hip")

5. TI ( knee* or patell* or menisc* or tibiofemoral ) OR AB ( knee* or patell* or menisc* or tibiofemoral )

6. TI ( (anterior or posterior) n2 "cruciate ligament*" OR ACL OR PCL) OR AB ( (anterior or posterior) n2 "cruciate ligament*" OR ACL OR PCL)

7. TI ( (medial or lateral) n2 "collateral ligament*" OR MCL OR LCL) OR AB ( (medial or lateral) n2 "collateral ligament*" OR MCL OR LCL)

8. TI ( hip* or acetabular or femor* or FAI or FAIS or groin* ) OR AB ( hip* or acetabular or femor* or FAI or FAIS or groin* )

9. S1 OR S2 OR S3 OR S4 OR S5 OR S6 OR S7 OR S8

10. (MH "Exercise+") OR (MH "Therapeutic Exercise+") OR (MH "Rehabilitation") OR (MH “Physical Therapy”) OR (MH “Gait”) OR (MH “Gait Training+”) OR (MH “Funcional Training”) OR (MH “Weight-bearing”)

11. (MH “Rehabilitation, Athletic”) OR (MH “Athletic Training”) OR (MH "Muscle Strengthening") OR (MH "Plyometrics") OR (MH "Closed Kinetic Chain Exercises") OR (MH "Open Kinetic Chain Exercises") OR (MH "Isokinetic Exercises") OR (MH "Isometric Exercises") OR (MH "Isotonic Exercises") OR (MH "Resistance Training")

12. (MH "Sports+") OR (MH "Muscle Strength")

13. TI ( exercis* or rehabilitat* or (musc* n3 strength*) or "closed kinetic chain" or "open kinetic chain" ) OR AB ( exercis* or rehabilitat* or (muscle n3 strength*) or "closed kinetic chain" or "open kinetic chain" )

14. TI ( (isotonic* or isokinetic* or isometric* or plyometric* or strength* or resist* or weight* or endurance or aerobic) n3 (train* or exercis*) ) OR AB ( (isotonic or isokinetic* or isometric* or strength* or resist* or weight* or endurance or aerobic) n3 (train* or exercis*) )

15. TI ( run* or jog* or walk* or bicycl* or cycl* or treadmill or marathon* or physical activit* ) OR AB ( run* or jog* or walk* or cycl* or treadmill or marathon or physical activit* )

16. TI ( weightbear* or weight-bear* or gait* or (joint* n3 (load* or force*)) ) OR AB ( weightbear* or weight-bear* or gait* or (joint* n3 (load* or force*)) )

17. S10 OR S11 OR S12 OR S13 OR S14 OR S15 OR S16

18. (MH "Magnetic Resonance Imaging+")

19. TI ( ("magnetic resonance" n3 imag*) or MR or MRI or T1* or T2* or dGEMRIC) OR AB ( ("magnetic resonance" n3 imag*) or MR or MRI or T1* or T2* or dGEMRIC )

20. S18 OR S19

21. (MH "Cartilage") OR (MH “Cartilage, Articular”) OR (MH "Collagen") OR (MH “Glycosaminoglycans”)

22. TI ( cartilag* or chondr* or collagen* or glycosaminoglycan* ) OR AB ( cartilag* or chondr* or collagen* or glycosaminoglycan* )

23. TI ( SHOMRI or WORMS or MOAKS or ACLOAS or BLOKS or KOSS ) OR AB ( SHOMRI or WORMS or MOAKS or ACLOAS or BLOKS or KOSS )

24. S21 OR S22 OR S23

25. S9 AND S17 AND S20 AND S24

SPORTSDiscus (via EBSCOhost)

1. (DE "KNEE" OR DE "ANTERIOR cruciate ligament" OR DE "MEDIAL collateral ligament (Knee)" OR DE "MENISCUS (Anatomy)" OR DE "PATELLA" OR DE "POSTEROLATERAL corner") OR (DE "PATELLOFEMORAL joint")

2. ((DE "KNEE injuries" OR DE "ANTERIOR cruciate ligament injuries" OR DE "POSTERIOR cruciate ligament injuries" OR DE "PATELLOFEMORAL joint injuries") OR (DE "MENISCUS injuries")) OR (DE "CHONDROMALACIA patellae")

3. DE "HIP joint"

4. (DE "HIP joint injuries") OR (DE "GROIN injuries" OR DE "GROIN pain")

5. TI ( knee* or tibial or tibiofemoral or menisc* ) OR AB ( knee* or tibial or tibiofemoral or menisc* ) OR KW ( knee* or tibial or tibiofemoral or menisc* )

6. TI ( patell* or PFP or PFPS or AKP ) OR AB ( patell* or PFP pr PFPS or AKP ) OR KW ( patell* or PFP pr PFPS or AKP )

7. TI ( "anterior cruciate ligament*" or ACL or "posterior cruciate ligament*" or PCL or "medial collateral ligament*" or MCL or "lateral collateral ligament*" or LCL ) OR AB ( "anterior cruciate ligament*" or ACL or "posterior cruciate ligament*" or PCL or "medial collateral ligament*" or MCL or "lateral collateral ligament*" or LCL ) OR KW ( "anterior cruciate ligament*" or ACL or "posterior cruciate ligament*" or PCL or "medial collateral ligament*" or MCL or "lateral collateral ligament*" or LCL )

8. TI ( hip* or femor* or acetabular or FAI or FAIS or "groin pain" ) OR AB ( hip* or femor* or acetabular or FAI or FAIS or "groin pain" ) OR KW ( hip* or femor* or acetabular or FAI or FAIS or "groin pain" )

9. S S1 OR S2 OR S3 OR S4 OR S5 OR S6 OR S7 OR S8

10. DE "EXERCISE" exp

11. DE "SPORTS" exp

12. DE "PHYSICAL training & conditioning" exp

13. TI ( exercis* or rehabilitat* or (musc* n3 strength*) ) OR AB ( exercis* or rehabilitat* or (muscle* n3 strength*) ) OR KW ( exercis* or rehabilitat* or (muscle* n3 strength*) )

14. TI ( (isotonic* or isometric* or isokinetic* or plyometric* or resist* or weight* or endurance or aerobic) n3 (train* or exercis*) ) OR AB ( (isotonic* or isometric* or isokinetic* or plyometric* or resist* or weight* or endurance or aerobic) n3 (train* or exercis*) ) OR KW ( (isotonic* or isometric* or isokinetic* or plyometric* or resist* or weight* or endurance or aerobic) n3 (train* or exercis*) )

15. TI ( run* or jog* or walk* or gait* or cycl* or bicycl* or treadmill or marathon or "physical activit*" ) OR AB ( run* or jog* or walk* or gait* or treadmill or marathon or "physical activit*" ) OR KW ( run* or jog* or walk* or gait* or treadmill or marathon or "physical activit*" )

16. DE "WEIGHT-bearing (Orthopedics)"

17. TI ( weight-bear* or weightbear* or (joint* n3 (load* or force*)) ) OR AB ( weight-bear* or weightbear* or (joint* n3 (load* or force*)) ) OR KW ( weight-bear* or weightbear* or (joint* n3 (load* or force*)) )

18. S10 OR S11 OR S12 OR S13 OR S14 OR S15 OR S16 OR S17

19. (DE "NUCLEAR magnetic resonance") OR (DE "MAGNETIC resonance imaging")

20. TI ( "magnetic resonance" n3 imag* or MR or MRI or T1* or T2* or dGEMRIC ) OR AB ( "magnetic resonance" n3 imag* or MR or MRI or T1* or T2* or dGEMRIC ) OR KW ( "magnetic resonance" n3 imag* or MR or MRI or T1* or T2* or dGEMRIC )

21. S20 OR S21

22. (DE "CARTILAGE" OR DE "CARTILAGE fractures" OR DE "CARTILAGE injuries") OR (DE "ARTICULAR cartilage")

23. TI ( cartilag* or chondr* or collagen* or glycosaminoglycan* ) OR AB ( cartilag* or chondr* or collagen* or glycosaminoglycan* ) OR KW ( cartilag* or chondr* or collagen* or glycosaminoglycan*)

24. TI ( SHOMRI or WORMS or MOAKS or ACLOAS or BLOKS or KOSS ) OR AB ( SHOMRI or WORMS or MOAKS or ACLOAS or BLOKS or KOSS ) OR KW ( SHOMRI or WORMS or MOAKS or ACLOAS or BLOKS or KOSS )

25. S23 OR S24 OR S25

COCHRANE LIBRARY

Search Hits

#1 MeSH descriptor: [Knee Joint] explode all trees

#2 MeSH descriptor: [Patellofemoral Joint] explode all trees

#3 MeSH descriptor: [Anterior Cruciate Ligament] explode all trees

#4 MeSH descriptor: [Menisci, Tibial] explode all trees

#5 (knee or patellofemoral or femoropatella or tibiofemoral or meniscus or "anterior cruciate ligament" or ACL):ti,ab,kw

#6 #1 or #2 or #3 or #4 or #5

#7 MeSH descriptor: [Hip Joint] explode all trees

#8 (hip or acetabular or femoral):ti,ab,kw

#9 MeSH descriptor: [Osteoarthritis, Knee] explode all trees

#10 MeSH descriptor: [Osteoarthritis, Hip] explode all trees

#11 MeSH descriptor: [Knee Injuries] explode all trees

#12 MeSH descriptor: [Hip Injuries] explode all trees

#13 MeSH descriptor: [Anterior Cruciate Ligament Injuries] explode all trees

#14 MeSH descriptor: [Tibial Meniscus Injuries] explode all trees

#15 MeSH descriptor: [Femoracetabular Impingement] explode all trees

#16 #6 or #7 or #8 or #9 or #10 or #11 or #12 or #13 or #14 or #15

#17 MeSH descriptor: [Exercise] explode all trees

#18 MeSH descriptor: [Exercise Therapy] explode all trees

#19 MeSH descriptor: [Exercise Movement Techniques] explode all trees

#20 MeSH descriptor: [Rehabilitation] explode all trees

#21 MeSH descriptor: [Muscle Strength] explode all trees

#22 (exercise or rehabilitation or muscle n3 strength):ti,ab,kw

#23 #17 or #18 or #19 or #20 or #21 or #22

#24 ("closed kinetic chain" or "open kinetic chain" or isometric or isokinetic or isotonic or plyometric):ti,ab,kw

#25 MeSH descriptor: [Resistance Training] explode all trees

#26 MeSH descriptor: [Weight Lifting] explode all trees

#27 (weight or resistance or strength) near/5 training or "physical activity"

#28 #24 or #25 or #26 or #27

#29 MeSH descriptor: [Endurance Training] explode all trees

#30 MeSH descriptor: [Running] explode all trees

#31 MeSH descriptor: [Jogging] explode all trees

#32 MeSH descriptor: [Bicycling] explode all trees

#33 (run or jog or bicycling or endurance near/5 training):ti,ab,kw

#34 #29 or #30 or #31 or #32 or #33

#35 MeSH descriptor: [Walking] explode all trees

#36 MeSH descriptor: [Gait] explode all trees

#37 MeSH descriptor: [Weight-Bearing] explode all trees

#38 (walking or gait or weight-bear or weightbear or (joint near/5 loading)):ti,ab,kw

#39 #35 or #36 or #37 or #38

#40 MeSH descriptor: [Magnetic Resonance Imaging] explode all trees

#41 MeSH descriptor: [Magnetic Resonance Spectroscopy] explode all trees

#42 ((magnetic resonance near/3 imaging) or MRI or MR or T1 or T2 or dGEMRIC):ti,ab,kw

#43 #40 or #41 or #42

#44 MeSH descriptor: [Cartilage] explode all trees

#45 MeSH descriptor: [Cartilage, Articular] explode all trees

#46 MeSH descriptor: [Collagen] explode all trees

#47 MeSH descriptor: [Glycosaminoglycans] explode all trees

#48 (cartilage or chondral or collagen or glycosaminoglycan):ti,ab,kw

#49 (SHOMRI or MOAKS or WORMS or BLOKS or ACLOAS or KOSS):ti,ab,kw

#50 #44 or #45 or #46 or #47 or #48 or #49

#51 #6 or #16

#52 #23 or #28 or #34 or #39

#53 #43 and #50 and #51 and #52

WEB OF SCIENCE

1. TS=(knee* or menisc* or “anterior cruciate ligament*” or ACL or “ posterior cruciate ligament*” or PCL or patell* or tibiofemoral or “medial collateral ligament” or MCL or “lateral collateral ligament” or LCL)

2. TS=(hip* or femor* or acetabular or FAI or FAIS or groin*)

3. #2 or #1

4. TS=(exercise* or rehabilitat* or musc* near/3 strength*)

5. TS=(run* or jog* or walk* or gait* or bicycl* or “physical activit*”)

6. TS=(joint near/3 (load* or force*))

7. TS=(weightbear* or “weight bear*”)

8. TS=((weight* or resist* or endurance or aerobic) near/3 (train* or exercis*))

9. TS=((isometric* or isokinetic* or isotonic* or plyometric*) near/3 (train* or exercis*))

10. #9 or #8 or #7 or #6 or #5 or #4

11. TS=(“magnetic resonance” near/3 imag* or MRI or MR or T1 or T2 or dGEMRIC)

12. TS=(cartilage* or chondr* or collagen* or glycosaminoglycan*)

13. TS=(SHOMRI or MOAKS or WORMS or BLOKS or ACLOAS or KOSS)

14. #13 0r #12

15. #14 and #11 and #10 and #3
