# Supplementary material for: Immediate and Delayed Effects of Joint Loading Activities on Knee and Hip Cartilage: A Systematic Review and Meta-analysis
Source: Sports Med Open. 2023 Jul 14;9:56. doi: 10.1186/s40798-023-00602-7 (PMC10348990; doi:10.1186/s40798-023-00602-7)
Supplement: Supplementary file 2 — Additional file 2: The modified Newcastle-Ottawa Quality Assessment Scale (NOS) for risk of bias assessment. [file 40798_2023_602_MOESM2_ESM.docx]

**Online Resource 2. Modified Newcastle-Ottawa Quality Assessment Scale (NOS) for risk of bias assessment.**

Wells, G. et al, 2011. The Newcastle-Ottawa Scale (NOS) for assessing the quality of nonrandomised studies in meta-analyses. *http://www.ohri.ca/programs/clinical_epidemiology/oxford.asp*^19^ .

NA=not applicable

| CRITERIA | ADDITIONAL EXPLANATION/ MODIFICATIONS |
| --- | --- |
| **Selection:**  1. Definition of cases (and controls if used) was…  Low risk of bias = a | **a)** = clearly described with inclusion/exclusion criteria for **cases** using **clinical definitions** provided by clinician, researcher, self-report or record linkage. (Can we reproduce the sample of participants?)  For example:  i) injury/surgical history,  ii) OA classification,  iii) training history.  **AND** clearly described with inclusion/exclusion criteria for **controls (if used)** using **healthy/uninjured definitions.**  **b)**= not described **OR** used minimal criteria for inclusion/exclusion. |
| **Selection:**  2. Representativeness of the cases (and controls if used) was…  Low risk of bias = a | **a)** = **truly** representative of the population that presents with the condition of interest and sourced from the **general population of interest**.  For example:  i) OA: clinical or radiographic OA inclusion criteria  ii) at risk of (early) OA: risk factors reported such as previous injury, FAIS, elevated BMI, family history etc.  iii) healthy: no history of index joint injury/surgery/symptom  iv) runners: training history  v) sedentary: no regular exercise.  **OR somewhat** representative of the population that presents with the condition of interest and sourced from **convenience sample**. (Can we reproduce the sample of participants?)  For example:  i) university,  ii) hospital,  iii) sports club.  **b)** = not described **OR** a potential selection bias existed |
| **Selection:**  3. Sample size was…  Low risk of bias = a | **a)** = 20 or more participants in total.  **b)**= less than 20 participants in total. |
| **Selection:**  4. Activity level/exercise prior to baseline MRI was…  Low risk of bias = a | **a) = controlled** if study (measuring quantitative/qualitative cartilage MRI outcomes) instructed participants to avoid high joint loading activities or included rest period.  For example:  i) no stairs/running/sport/heavy lifting during the day before **OR** x hours before MRI  ii) rest period sitting/supine immediately before MRI  **b)** = **not controlled** if study (measuring quantitative/qualitative cartilage MRI outcomes) did not report any participant instructions to avoid high joint loading activities or include a rest period.  **N/A** = study reporting semi-quantitative (morphological) cartilage outcome |
| **Comparability:**  5. Comparability of cases and controls on the basis of the design or analysis …  Low risk of bias = a | **a) =** existed if study cases were a priori matched with controls for at least one covariate, or confounding controlled for in statistical analysis.  Covariate examples:  i) age,  ii) BMI,  iii) sex  **b)** = would not exist if statements comprised only that no differences between groups existed or that differences were not statistically significant (as these are not sufficient for establishing comparability)  **c)** = was not controlled in design or analysis and no confounders acknowledged  **N/A** = no comparator group used |
| **Outcomes**:  6. Ascertainment of MRI outcomes by assessor who was…  Low risk of bias = a | **a)** = blind to pre-/post-MRI timepoint **AND** blind to case/control status (if case /control study)  **b)** = blind only to pre-/post-MRI status **OR** only to case/control status (if case /control study)  **c)** = not blind to either pre-/post-MRI timepoint or case/control status (if case /control study) **OR** unable to determine |
| **Outcomes**:  7. Reliability and qualification of MRI assessors…  Low risk of bias = a | **a) =** was described reporting profession and experience of the MRI assessor(s) **AND** reliability was established.  For example:  i) musculoskeletal radiologist **AND** reliability established,  ii) single trained observer with amount of training/experience described **AND** reliability established  iii) two or more trained observers **AND** reliability established.  iv) fully automated assessment with no assessor required  **b) =** was not described in terms of profession and experience of the MRI assessor and **NO** reliability established |
| **Outcomes**:  8. Reliability/validity of MRI outcome measures were…  Low risk of bias = a | **a) =** clearly described **AND** are reproducibility, validity or reliability was referenced or demonstrated. For example:  i) referenced other article(s) which found same outcome measure to be valid and reliable  ii) demonstrated the outcome measure(s) are reproducible, valid or reliable  **b) =** not explained in reproducible detail **AND** validity and reliability not demonstrated/referenced. |
| **Outcomes:**  9. Adequacy of follow-up was…  Low risk of bias = a | **a)** = adequately reported and minimal loss to follow-up.  For example:  i) <15% loss to follow-up **AND** those lost to follow-up are described (i.e. age, sex, or BMI did not differ from the rest of the cohort etc.). Stating the reasons why they were lost to follow-up is not sufficient.  ii) <5% loss to follow-up and no description of those lost.  **b)** = not explicitly stated with number of participants lost to follow-up **OR** characteristics of those lost to follow-up were not described. |
