# Supplementary material for: Immediate and Delayed Effects of Joint Loading Activities on Knee and Hip Cartilage: A Systematic Review and Meta-analysis
Source: Sports Med Open. 2023 Jul 14;9:56. doi: 10.1186/s40798-023-00602-7 (PMC10348990; doi:10.1186/s40798-023-00602-7)
Supplement: Supplementary file 3 — Additional file 3: Risk of bias assessment using the NOS. [file 40798_2023_602_MOESM3_ESM.docx]

**Online Resource 3.** Risk of bias using the Newcastle-Ottawa Scale

| **Newcastle-Ottawa**  **Scale items** | | **Definition of cases (and controls if used)** | **Representativeness**  **of the cases (and controls if used)** | **Sample size is a≥20**  **or b<20** | **Activity level prior**  **to baseline MRI controlled** | **Comparability of**  **cases and controls**  **on the basis of the**  **design or analysis** | **Ascertainment of**  **MRI outcomes** | **Qualification of**  **MRI scan assessors** | **MRI outcome measures** | **Adequacy of**  **follow-up** |
| --- | --- | --- | --- | --- | --- | --- | --- | --- | --- | --- |
| **Study** | **Year** | **Selection 1** | **Selection 2** | **Selection 3** | **Compar-ability 1** | **Compar-ability 2** | **Outcome 1** | **Outcome 2** | **Outcome 3** | **Outcome 4** |
| **Chen et al. [65]** | **2017** | **low** | **high** | **low** | **low** | **n/a** | **low** | **high** | **high** | **high** |
| **Collins et al. [39]** | **2018** | **low** | **high** | **high** | **low** | **high** | **high** | **high** | **low** | **high** |
| **Cotofana et al. [40]** | **2011** | **low** | **low** | **low** | **low** | **n/a** | **low** | **high** | **low** | **low** |
| **Crook et al. [41]** | **2020** | **low** | **high** | **high** | **low** | **n/a** | **high** | **high** | **low** | **high** |
| **Cutcliffe et al. [42]** | **2020** | **low** | **high** | **high** | **low** | **n/a** | **high** | **high** | **low** | **high** |
| **Eckstein et al. [45]** | **1999** | **high** | **high** | **high** | **low** | **n/a** | **high** | **high** | **low** | **low** |
| **Eckstein et al. [44]** | **2000** | **high** | **low** | **high** | **low** | **n/a** | **high** | **high** | **low** | **high** |
| **Eckstein et al. [43]** | **2005** | **high** | **high** | **low** | **low** | **low** | **high** | **high** | **low** | **high** |
| **Farrokhi et al. [46]** | **2011** | **low** | **low** | **low** | **low** | **low** | **high** | **high** | **high** | **high** |
| **Gatti et al. [47]** | **2017** | **low** | **low** | **high** | **low** | **n/a** | **high** | **high** | **low** | **high** |
| **Hatcher et al.[66]** | **2017** | **high** | **high** | **high** | **low** | **n/a** | **high** | **high** | **low** | **high** |
| **Hesper et al. [76]** | **2017** | **high** | **high** | **high** | **low** | **n/a** | **high** | **low** | **high** | **low** |
| **Ho et al. [48]** | **2019** | **low** | **high** | **high** | **low** | **low** | **low** | **high** | **low** | **high** |
| **Horng et al. [19]** | **2015** | **high** | **high** | **high** | **low** | **n/a** | **low** | **low** | **low** | **high** |
| **Hudelmaier et al. [49]** | **2001** | **high** | **high** | **low** | **high** | **high** | **high** | **high** | **low** | **low** |
| **Jogi et al. [50]** | **2021** | **high** | **high** | **high** | **high** | **n/a** | **high** | **low** | **high** | **low** |
| **Lad et al. [51]** | **2016** | **high** | **high** | **high** | **low** | **n/a** | **high** | **high** | **low** | **high** |
| **Lange et al. [67]** | **2017** | **high** | **high** | **high** | **high** | **n/a** | **high** | **high** | **high** | **low** |
| **Lange et al. [52]** | **2019** | **high** | **high** | **high** | **low** | **n/a** | **high** | **high** | **low** | **low** |
| **Marsh et al. [53]** | **2013** | **low** | **high** | **low** | **low** | **low** | **high** | **high** | **low** | **low** |
| **Mayerhoefer et al. [68]** | **2010** | **low** | **high** | **high** | **low** | **n/a** | **low** | **high** | **low** | **high** |
| **Nag et al. [69]** | **2004** | **high** | **high** | **low** | **low** | **n/a** | **high** | **high** | **low** | **high** |
| **Niehoff et al. [54]** | **2011** | **low** | **high** | **low** | **high** | **n/a** | **low** | **high** | **low** | **high** |
| **Nishii et al. [55]** | **2008** | **high** | **high** | **low** | **high** | **n/a** | **high** | **high** | **low** | **high** |
| **Nishii et al. [75]** | **2010** | **low** | **high** | **low** | **low** | **low** | **high** | **low** | **low** | **high** |
| **Owusu et al. [56]** | **2018** | **high** | **high** | **high** | **low** | **low** | **high** | **high** | **low** | **high** |
| **Paranjape et al. [57]** | **2019** | **high** | **high** | **high** | **low** | **n/a** | **high** | **high** | **low** | **high** |
| **Schoenbauer et al. [70]** | **2015** | **high** | **high** | **high** | **low** | **high** | **high** | **high** | **high** | **high** |
| **Schütz et al. [58]** | **2022** | **high** | **high** | **high** | **low** | **high** | **high** | **low** | **high** | **low** |
| **Sitoci et al. [59]** | **2012** | **high** | **high** | **high** | **low** | **n/a** | **high** | **high** | **low** | **high** |
| **Souza et al. [71]** | **2010** | **low** | **low** | **low** | **low** | **low** | **low** | **high** | **low** | **low** |
| **Souza et al. [72]** | **2014** | **low** | **low** | **low** | **low** | **low** | **high** | **high** | **low** | **low** |
| **Stehling et al. [74]** | **2012** | **low** | **low** | **low** | **low** | **low** | **high** | **high** | **low** | **low** |
| **Subburaj et al. [73]** | **2012** | **high** | **high** | **low** | **low** | **low** | **high** | **high** | **low** | **high** |
| **Sutter et al. [61]** | **2015** | **high** | **high** | **high** | **low** | **n/a** | **high** | **high** | **low** | **high** |
| **Sutter et al. [60]** | **2019** | **high** | **high** | **high** | **low** | **n/a** | **high** | **high** | **low** | **low** |
| **Tamayo et al. [62]** | **2022** | **low** | **high** | **high** | **low** | **high** | **high** | **high** | **low** | **high** |
| **Van Ginckel et al. [63]** | **2013** | **low** | **low** | **low** | **low** | **low** | **high** | **low** | **low** | **low** |
| **Verschueren et al. [31]** | **2017** | **high** | **high** | **low** | **high** | **n/a** | **high** | **high** | **low** | **high** |
| **Wang et al. [64]** | **2015** | **high** | **high** | **high** | **low** | **n/a** | **high** | **high** | **low** | **low** |

| **low risk**  **(>50% applicable items low risk)** | **high risk**  **(≤50% applicable items low risk)** | **not applicable** |
| --- | --- | --- |
