# Supplementary material for: Immediate and Delayed Effects of Joint Loading Activities on Knee and Hip Cartilage: A Systematic Review and Meta-analysis
Source: Sports Med Open. 2023 Jul 14;9:56. doi: 10.1186/s40798-023-00602-7 (PMC10348990; doi:10.1186/s40798-023-00602-7)
Supplement: Supplementary file 4 — Additional file 4: GRADE Certainty of the evidence assessment. [file 40798_2023_602_MOESM4_ESM.docx]

**Online Resource 4: Certainty of the evidence, MRI cartilage changes immediately after joint loading activities^1^.**

| Datasets (n) | Total in analysis  (n) | Joint loading activity | Risk of bias^2^ | Inconsist-ency | Indirect-ness | Imprecision | Public-ation bias | Certainty |
| --- | --- | --- | --- | --- | --- | --- | --- | --- |
| Healthy adults | | | | | | | | |
| *Immediate effects of joint loading activities on knee cartilage MRI thickness and volume* | | | | | | | | |
| Weightbearing femoral | | | | | | | | |
| 4 | 46 | Sim. stand | Very serious | Not serious | Not serious | Not serious | Serious | Very low |
| 4 | 34 | Walk | Serious | Not serious | Not serious | Not serious | Serious | Very low |
| 3 | 38 | Knee bends | Serious | Very serious | Not serious | Not serious | Serious | Very low |
| 3 | 32 | Hop/jump | Very serious | Not serious | Not serious | Not serious | Serious | Very low |
| Tibial | | | | | | | | |
| 4 | 46 | Sim. stand | Very serious | Very serious | Not serious | Not serious | Serious | Very low |
| 5 | 45 | Walk | Very serious | Serious | Not serious | Not serious | Serious | Very low |
| 4 | 55 | Knee bends | Serious | Very serious | Not serious | Not serious | Serious | Very low |
| 3 | 32 | Hop/jump | Very serious | Very serious | Not serious | Not serious | Serious | Very low |
| Patellar | | | | | | | | |
| 6 | 106 | Knee bends | Very serious | Very serious | Not serious | Not serious | Serious | Very low |
| 2 | 22 | Hop/jump | Very serious | Not serious | Not serious | Not serious | Serious | Very low |
| *Immediate effects of joint loading activities on knee cartilage MRI composition* | | | | | | | | |
| Weightbearing femoral | | | | | | | | |
| 4 | 150 | Sim. stand | Serious | Very serious | Not serious | Not serious | Serious | Very low |
| 2 | 25 | cycle | Very serious | Not serious | Not serious | Not serious | Serious | Very low |
| Tibial | | | | | | | | |
| 4 | 150 | Sim. stand | Serious | Not serious | Not serious | Not serious | Serious | Very low |
|  |  |  |  |  |  |  |  |  |
| 2 | 25 | Cycle | Very serious | Not serious | Not serious | Not serious | Serious | Very low |
| Adults with or at risk of OA | | | | | | | | |
| *Immediate effects of joint loading activities on knee cartilage MRI thickness and volume* | | | | | | | | |
| Weightbearing femoral | | | | | | | | |
| 2 | 19 | Sim. stand | Not serious | Not serious | Not serious | Not serious | Serious | Very low |
| 3 | 24 | Walk | Serious | Serious | Not serious | Not serious | Serious | Very low |
| Tibial | | | | | | | | |
| 2 | 19 | Sim. stand | Not serious | Not serious | Not serious | Not serious | Serious | Very low |
| 2 | 15 | Walk | Very serious | Very serious | Not serious | Not serious | Serious | Very low |

^1^All studies observational and therefore rated low certainty,

^2^Risk of bias assessment included in separate table (Online Resource 2),

MRI=magnetic resonance imaging, Sim. Stand=simulated standing (50% of body-weight applied within the scanner)

**Grade Working Group grades of certainty in the evidence:**

**High certainty**: We are very confident that the true effect lies close to that of the estimate of the effect.

**Moderate certainty**: We are moderately confident in the effect estimate: The true effect is likely to be close to the estimate of the effect, but there is a possibility that it is substantially different.

**Low certainty**: Our confidence in the effect estimate is limited: The true effect may be substantially different from the estimate of the effect.

**Very low certainty**: We have very little confidence in the effect estimate: The true effect is likely to be substantially different from the estimate of effect.

**Risk of bias:**

**Serious**: More than 25% of participants from studies with high risk of bias

**Very serious**: More than 75% or participants from studies with high risk of bias

**Inconsistency:**

**Serious**:I^2^>50% indicates substantial heterogeneity

**Very serious**: I^2^>75% indicates considerable heterogeneity
