# Supplementary material for: Immediate and Delayed Effects of Joint Loading Activities on Knee and Hip Cartilage: A Systematic Review and Meta-analysis
Source: Sports Med Open. 2023 Jul 14;9:56. doi: 10.1186/s40798-023-00602-7 (PMC10348990; doi:10.1186/s40798-023-00602-7)
Supplement: Supplementary file 7 — Additional file 7: Summary of meta-regression analyses exploring associations between sex, age, and activity duration/repetitions and cartilage thickness and volume changes. [file 40798_2023_602_MOESM7_ESM.docx]

**Online Resource 7. Summary of meta-regression analyses.**

| Cartilage thickness or volume changes: after activity, by region | For every… | Cartilage thickness or volume: [-] decreases/  decrease is reduced | 95% confidence interval | p value |
| --- | --- | --- | --- | --- |
| Knee bends:  Patellar cartilage | **10 repetition increase** | **-2.0%** | **-3.3 to -0.6** | **0.009*** |
|  | **10% increase in female participants** | **-0.3%** | **-0.7 to 0.1** | **0.143** |
|  | **10 year increase in mean age** | **0.9%** | **-0.2 to 2.0** | **0.097** |
| Knee bends:  Weightbearing femoral cartilage | **10 repetition increase** | **-2.2%** | **-8.3 to 4.0** | **0.141** |
|  | **10% increase in female participants** | **-2.0%** | **-2.2 to 1.7** | **0.410** |
|  | **10 year increase in mean age** | **Insufficient observations^1^** | | |
| Knee bends:  Tibial cartilage | **10 repetition increase** | **-1.8%** | **-3.9 to 0.0** | **0.068** |
|  | **10% increase in female participants** | **-0.8%** | **-8.1 to 6.5** | **0.686** |
|  | **10 year increase in mean age** | **1.1%** | **-1.5 to 1.7** | **0.547** |
| Walking:  Patellar cartilage | **Insufficient observations all variables** | | | |
| Walking:  Weightbearing femoral cartilage | **10 minute increase in duration** | **-0.1%** | **-1.4 to 1.4** | **0.980** |
|  | **10% increase in female participants** | **-0.2%** | **-2.2 to 1.9** | **0.774** |
|  | **10 year increase in mean age** | **0.1%** | **-3.3 to 3.5** | **0.884** |
| Walking:  Tibial cartilage | **10 minute increase in duration** | **-1.4%** | **-3.6 to 0.8** | **0.142** |
|  | **10% increase in female participants** | **-0.5%** | **-1.7 to 0.6** | **0.236** |
|  | **10 year increase in mean age** | **1.4%** | **-2.0 to 4.7** | **0.283** |
| Simulated stand^2^:  Weightbearing femoral cartilage | **10 minute increase in duration** | **0.4%** | **-5.2 to 5.9** | **0.529** |
|  | **10% increase in female participants** | **0.5%** | **-5.9 to 6.9** | **0.506** |
|  | **10 year increase in mean age** | **0.6%** | **-7.7 to 9.0** | **0.505** |
| Simulated stand^2^:  Tibial cartilage | **10 minute increase in duration** | **2.7%** | **-1.5 to 2.1** | **0.305** |
|  | **10% increase in female participants** | **0.9%** | **-3.1 to 3.3** | **0.772** |
|  | **10 year increase in mean age** | **1.5%** | **-40.6 to 4.4** | **0.730** |
| Hop/Jump:  all regions | **Insufficient observations all variables** | | | |
| Cycle:  all regions | **Insufficient observations all variables** | | | |

***Significance p<0.05,**

**^1^Eckstein 2005 mean age not reported,**

**^2^Simulated stand=50% body weight applied within the MRI scanner.**
